# Global, regional, and national burden of acute glomerulonephritis in children and adolescents: 1990–2021 analysis and future projections

**DOI:** 10.3389/fpubh.2025.1677679

**Published:** 2025-12-03

**Authors:** Ming Liu, Jing Liao, Yunpeng Gou, Ping Yang

**Affiliations:** 1 Department of Pediatric Surgery, Suining Central Hospital, Suining, Sichuan, China; 2 Department of Respiratory Medicine, Children’s Hospital of Chongqing Medical University, Chongqing, China

**Keywords:** acute glomerulonephritis (AGN), disability-adjusted life years (DALYs), epidemiology, socio-demographic index (SDI), public health interventions

## Abstract

**Background:**

Acute glomerulonephritis (AGN) remains a significant issue in global health, yet its burden among children and adolescents has not been sufficiently characterized. This study aims to systematically estimate the global, regional, and national burden of AGN, as well as its temporal trends from 1990 to 2021.

**Methods:**

This study utilized data from the Global Burden of Disease (GBD) 2021 study, focusing on incidence rates, disability-adjusted life years (DALYs), and mortality among individuals under 20 years of age. Estimated annual percentage changes (EAPCs) were employed to assess temporal trends, and statistical analyses were conducted to examine correlations with the Socio-Demographic Index (SDI). Additionally, we performed decomposition and inequality analyses, along with Bayesian Age-Period-Cohort (BAPC) analyses, to evaluate trends and distributions related to the burden of AGN.

**Results:**

From 1990 to 2021, the global burden of AGN among children and adolescents remained significantly high. In 2021, there were approximately 170,584 new cases, representing a decrease compared to earlier years, with an age-standardized incidence rate (ASIR) of 6.47 per 100,000 (95% UI: 4.79–8.45). The EAPC for incidence showed a declining trend of −1.03% (95% CI: −1.15, −0.92). The total number of DALYs associated with AGN was 59,588.50 (95% UI: 32,925.73–79,649.94), with an age-standardized DALY rate of 2.26 per 100,000 (95% UI: 1.25–3.02), demonstrating a significant decline represented by an EAPC of −4.33% (95% CI: −4.46, −4.21). Gender differences were minimal; however, disparities across SDI regions were pronounced, with middle SDI regions exhibiting the highest incidence rate at 8.87 per 100,000, and increasing incidence rates observed in lower-middle and low SDI regions. Notably, China reported the highest number of cases, followed by Brazil and India. Furthermore, absolute inequality among SDI countries improved from 1990 to 2021, while relative inequality intensified during the same period.

**Conclusion:**

This study underscores the substantial and evolving burden of AGN among children and adolescents, highlighting the urgent need for targeted public health strategies and interventions to address disparities in disease burden and improve health outcomes in vulnerable populations.

## Introduction

Acute glomerulonephritis (AGN) is a disease characterized by glomerular inflammation, typically manifesting as hematuria and proteinuria. It is particularly prevalent among children and in resource-limited regions, imposing a significant health burden (1). AGN has a high incidence in children and arises from multiple etiologies, including infections, immune responses, and genetic factors (1). Various infectious agents can trigger AGN, with Group A Streptococcus from pharyngeal or cutaneous infections being the most common bacterial cause (2, 3). Additionally, other pathogens, such as Staphylococcus, specific viruses, and parasites, can also induce AGN (4). This inflammatory kidney disease primarily damages the glomeruli, thereby reducing their blood filtration capacity. Childhood post-streptococcal acute glomerulonephritis (PSAGN) remains a significant risk factor for chronic kidney disease (5). The Global Burden of Disease (GBD) study estimates approximately 722,244 new cases globally in 2019, with a higher incidence in low-resource regions (6, 7), indicating that AGN continues to pose a significant global public health challenge.

The health burden of AGN remains consistently high, particularly in economically disadvantaged areas. The scarcity of effective medical resources and management strategies exacerbates the epidemiological characteristics of this disease in certain developing countries (8). While enhanced living conditions and increased access to antibiotics have reduced AGN incidence in developed nations, the burden of the disease remains significant in low- and middle-income countries (9, 10).

Despite the recognized global significance of AGN, comprehensive epidemiological data specifically targeting pediatric and adolescent populations remain notably sparse. A representative study (1) in central Australia documented a post-streptococcal AGN incidence rate of 228.7 per 100,000 person-years, highlighting regional variability. Existing epidemiological evidence indicates that AGN incidence predominantly peaks among children aged 5–14 years (11), attributable to multifactorial determinants including increased pathogen exposure within school settings, immunological immaturity, and heightened susceptibility to streptococcal infections during this developmental stage (12, 13). Nevertheless, contemporary research has yet to systematically quantify the disease burden among individuals under 20 years across diverse sociodemographic contexts, nor comprehensively analyze longitudinal epidemiological trends within this vulnerable population.

This study aims to address the critical research gap by providing a comprehensive epidemiological analysis of AGN burden among children and adolescents, utilizing data from the GBD 2021 study. We systematically assess the incidence and disability-adjusted life years (DALYs) for individuals under 20 years across 204 countries and territories over a 31-year period from 1990 to 2021. By meticulously examining age-standardized rates, longitudinal epidemiological trends, and multifaceted sociodemographic determinants, this research offers nuanced comparative insights into pediatric AGN burden across diverse geographical regions and developmental contexts. The findings will strategically inform targeted prevention strategies, optimize resource allocation, and guide evidence-based policy interventions to mitigate the burden of this potentially preventable renal pathology among global youth populations.

## Methods

### Data source and study population

The Institute for Health Metrics and Evaluation at the University of Washington leads the GBD study, which is widely regarded as one of the most comprehensive epidemiological initiatives globally. This systematic effort quantifies health losses from 371 diseases and injuries across 204 countries and territories, offering an in-depth assessment of global health challenges (14). Data for this analysis were sourced from the Global Burden of Disease Study 2021 (GBD 2021) via the Global Health Data Exchange (GHDx) query tool from the Institute for Health Metrics and Evaluation.^1^ We extracted estimates for acute glomerulonephritis using the following specific query parameters: cause = “acute glomerulonephritis” (GBD cause ID: 588), measure = “Incidence/Deaths/Disability-adjusted life years (DALYs),” metric = “Number/Rate” and year = “1990–2021,” and the analysis focused on the population under 20 years stratified into four age groups (under 5 years, 5–9 years, 10–14 years, and 15–19 years) across 204 countries and territories and 21 GBD regions. Age-standardized rates were calculated using the GBD standard population for individuals under 20 years of age. This research adhered to the Guidelines for Accurate and Transparent Health Estimates Reporting. The AGN burden in GBD analyses is evaluated using three primary metrics: mortality, incidence, and DALYs. DALYs encompass both premature mortality (Years of Life Lost, YLL) and non-fatal health impairment (Years Lived with Disability, YLD).

### Socio-demographic index

The Socio-Demographic Index (SDI), a composite measure that integrates income per capita, average educational attainment, and fertility rate (15), was used to evaluate the association between socioeconomic development and the AGN burden. With values ranging from 0 to 1, countries were categorized into five quintiles: low (0–0.47), low-middle (0.48–0.59), middle (0.60–0.69), high-middle (0.70–0.80), and high (0.81–1.0).

### Statistical analysis

Primary metrics included age-standardized incidence rates (ASIRs) and DALY rates, expressed per 100,000 population along with 95% uncertainty intervals (UIs). We calculated the age-standardized incidence rates (ASIR) and age-standardized DALYs rates (ASDR) based on the GBD World Standard Population using the following formula: $\mathrm{ASR}=\frac{\sum_{i=1}^{N}\;a_{i}w_{i}}{\sum_{i=1}^{N}\;w_{i}}$ , where $a_{i}$ is the ASR in the ith age group and $w_{i}$ represents the number of people (or the weight) in the same age group among the GBD standard population. N is the number of age groups (16). Temporal trends in disease burden were assessed using estimated annual percentage changes (EAPCs) derived from a regression model applied to age-standardized rates: *y* = *α* + *βx*, where y represents ln(ASIR) and x represents the year. The EAPC was calculated as 100 × (*e^β^* − 1) with 95% confidence intervals (CIs) (16, 17). Trends were considered statistically significant when both the EAPC and its 95% CI excluded zero. The association between the SDI and disease burden was assessed using Locally Estimated Scatterplot Smoothing curves (span = 0.75) and Spearman’s rank correlation coefficient.

Frontier analysis identified healthcare system efficiency by establishing the theoretical minimum AGN burden achievable at each SDI level using 2021 data. The efficiency gap (effective difference) was calculated as the difference between the observed DALY rates and the frontier value. Countries on or near the frontier were classified as optimally performing relative to their level of development (18).

Health inequality was quantified using multiple metrics: the Gini and concentration indices measured overall inequality in burden distribution across nations; the decomposable Theil index assessed the contributions of regional and country-level disparities; Lorenz curves visualized the cumulative burden against population proportions; and slope index of inequality (SII) quantified absolute disparities along the SDI gradient (18). All indices were bootstrapped with 1,000 iterations to calculate 95% CIs. These analyses were conducted using the ‘reldist’ package (version 1.7–2) for Gini coefficient, the ‘IC2’ package (version 1.0–1) for concentration indices, and custom R functions for Theil index and SII calculations.

Bayesian Age–Period–Cohort (BAPC) specification and forecasting. To project future AGN burden, we used a BAPC model implemented via integrated nested Laplace approximations (INLA). Ages were grouped into 0–4, 5–9, 10–14, and 15–19 years; periods were annual (1990–2050); cohorts were implied by age and period. We modeled log-rates with smoothing/penalty structures: age = second-order random walk (RW2), period = first-order random walk (RW1), cohort = second-order random walk (RW2), plus an i.i.d. over-dispersion term. Precision hyperpriors followed log-Gamma (1, 0.00005) for age/period/cohort and log-Gamma (1, 0.005) for over-dispersion. Population denominators and standard weights were used where applicable to obtain age-standardized projections. Posterior predictive means and standard deviations were extracted, and 95% predictive intervals were computed as mean ± 1.96 × SD. Internal optimization diagnostics reported no failed approximations or divergent modes.

We evaluated model adequacy using posterior predictive checks by comparing observed under-20 aggregated rates—constructed from age-specific counts and denominators with fixed age weights held constant over time—with the model’s posterior predictive distributions for 1990–2021, and summarized predictive performance with RMSE, MAE, MAPE, and 95% predictive coverage. This BAPC approach is widely used in epidemiology and biostatistics and integrates both sample information and prior knowledge to obtain robust and reliable parameter estimates (19–21).

All statistical analyses and visualizations were performed using R software (version 4.2.3). Specific R packages, including map, ggplot2, and dplyr, for example, were utilized in this analysis. Detailed R scripts for all analyses are provided in the Supplementary materials.

### Ethics statement

For GBD studies, the Institutional Review Board of the University of Washington reviewed and approved a waiver of informed consent^2^ (14, 16).

## Results

### Global disease burden of AGN in children and adolescents from 1990 to 2021

Globally, in 2021, the burden of AGN in children and adolescents remained significant, with an estimated 170,584.89 (95% UI: 126,380.92–222,733.53) incident cases. The number of deaths was estimated at 722.35 (95% UI: 397.94–968.63), while the number of DALYs totaled 59,588.50 (95% UI: 32,925.73–79,649.94) (Tables 1, 2; Supplementary Table S1). The ASIR of AGN in this demographic was 6.47 (95% UI, 4.79–8.45) per 100,000 population in 2021, and the EAPC showed a decreasing trend from 1990 to 2021 (EAPC = −1.03, 95% CI: −1.15, −0.92, Table 1). Worldwide, the age-standardized death rate (ASDR) was relatively low, at 0.03 (95% UI, 0.02–0.04) per 100,000 population, exhibiting a substantial decreasing trend from 1990 to 2021 (EAPC = −4.34, 95% CI: −4.46, −4.22, Supplementary Table S1). The age-standardized DALY rate in 2021 was 2.26 (95% UI, 1.25–3.02) per 100,000 population, with an EAPC reflecting a decline of 4.33% from 1990 to 2021 (EAPC = −4.33, 95% CI: −4.46, −4.21, Table 2). Gender disparities were modest, while significant variations were observed across socioeconomic development index (SDI) regions. Middle SDI regions had the highest incidence rate in 2021, at 8.87 per 100,000, which was 1.74 times higher than the rate in high SDI regions (5.10 per 100,000). In contrast, low-middle and low SDI regions exhibited concerning upward trends (EAPCs of 0.42 and 0.07%, respectively, Table 1).

**Table 1:** Number of incidence and age-standardized incidence rates per 100,000 population of acute glomerulonephritis among children and adolescents in 1990 and 2021 and temporal trends.

Characteristics	1990		2021		1990–2021
	Incidence cases no. (95%UI)	Rate/100,000	Incidence cases no. (95%UI)	Incidence rate/100,000 no. (95%UI)	EAPC no. (95%CI)
Overall	204191.13 (142133.11, 270768.37)	9.04 (6.29, 11.99)	170584.89 (126380.92, 222733.53)	6.47 (4.79, 8.45)	−1.03 (−1.15, −0.92)
Sex					
Male	101658.10 (70044.76, 135045.21)	8.78 (6.05, 11.67)	85893.98 (64036.92, 111667.87)	6.32 (4.71, 8.22)	−0.99 (−1.12, −0.87)
Female	102533.04 (71891.17, 136375.30)	9.31 (6.53, 12.38)	84690.91 (62305.91, 110491.78)	6.63 (4.88, 8.65)	−1.07 (−1.18, −0.96)
Socio-demographic factor					
High SDI	16056.83 (11105.83, 21638.45)	6.39 (4.42, 8.61)	11868.29 (8586.19, 15844.06)	5.10 (3.69, 6.81)	−0.59 (−0.77, −0.42)
High-middle SDI	49272.17 (31469.68, 67387.83)	13.31 (8.50, 18.20)	21992.73 (15709.78, 29380.87)	7.25 (5.18, 9.69)	−2.13 (−2.34, −1.92)
Middle SDI	95489.70 (67085.89, 126080.57)	12.49 (8.77, 16.49)	66448.18 (50174.59, 86125.46)	8.87 (6.70, 11.50)	−1.02 (−1.19, −0.86)
Low-middle SDI	32008.88 (23791.12, 41670.18)	5.42 (4.03, 7.05)	45931.27 (34251.22, 59303.24)	6.01 (4.48, 7.76)	0.42 (0.33, 0.51)
Low SDI	11175.30 (7982.94, 14671.66)	4.00 (2.86, 5.25)	24176.92 (16819.30, 32292.80)	4.14 (2.88, 5.53)	0.07 (0.05, 0.09)
Region					
Andean Latin America	1044.04 (682.07, 1461.08)	5.51 (3.60, 7.71)	1457.77 (986.56, 2025.83)	6.16 (4.17, 8.56)	0.38 (0.30, 0.46)
Australasia	217.88 (145.89, 303.28)	3.47 (2.33, 4.83)	257.01 (176.31, 356.94)	3.41 (2.34, 4.73)	−0.19 (−0.36, −0.01)
Caribbean	906.59 (584.64, 1248.28)	6.00 (3.87, 8.27)	955.31 (614.98, 1327.22)	6.26 (4.03, 8.70)	0.11 (0.04, 0.17)
Central Asia	2965.01 (1863.96, 4088.09)	9.39 (5.90, 12.95)	3350.60 (2108.32, 4665.95)	9.68 (6.09, 13.48)	0.05 (−0.12, 0.23)
Central Europe	4217.43 (2883.39, 5686.00)	10.74 (7.34, 14.48)	2391.40 (1640.76, 3236.39)	10.15 (6.97, 13.74)	−0.22 (−0.27, −0.16)
Central Latin America	5995.06 (3945.92, 8320.30)	7.26 (4.78, 10.07)	5655.64 (3655.82, 7859.98)	6.63 (4.29, 9.22)	−0.61 (−0.73, −0.49)
Central Sub-Saharan Africa	1680.32 (1113.57, 2271.87)	5.42 (3.59, 7.33)	3952.05 (2539.14, 5430.99)	5.37 (3.45, 7.38)	−0.04 (−0.05, −0.02)
East Asia	91129.62 (59333.17, 123447.31)	19.80 (12.89, 26.83)	32583.51 (24215.03, 42767.92)	9.45 (7.02, 12.40)	−2.39 (−2.59, −2.19)
Eastern Europe	6641.87 (4975.67, 8479.87)	9.87 (7.40, 12.60)	4123.42 (3090.69, 5330.18)	8.93 (6.70, 11.55)	−0.72 (−0.85, −0.58)
Eastern Sub-Saharan Africa	5783.51 (4175.61, 7587.97)	5.22 (3.77, 6.84)	11533.73 (8073.31, 15456.95)	5.07 (3.55, 6.79)	−0.18 (−0.23, −0.13)
High-income Asia Pacific	5705.13 (4076.60, 7647.00)	11.34 (8.10, 15.19)	4771.09 (3521.33, 6339.63)	15.50 (11.44, 20.59)	1.61 (1.26, 1.96)
High-income North America	1279.38 (820.94, 1809.87)	1.57 (1.00, 2.21)	1074.85 (840.65, 1347.94)	1.20 (0.94, 1.51)	−0.94 (−1.03, −0.85)
North Africa and Middle East	9115.30 (6192.82, 12176.31)	5.16 (3.50, 6.89)	14943.19 (10161.18, 20009.74)	6.32 (4.30, 8.46)	0.72 (0.70, 0.74)
Oceania	373.84 (239.65, 511.15)	11.10 (7.12, 15.18)	695.04 (450.42, 951.98)	10.88 (7.05,14.91)	−0.07 (−0.16, 0.01)
South Asia	13659.12 (10454.97, 17507.03)	2.52 (1.93, 3.23)	18300.00 (13903.36, 23491.60)	2.68 (2.03,3.44)	0.18 (0.13, 0.24)
Southeast Asia	37466.08 (27313.16, 49398.58)	17.04 (12.42, 22.46)	30054.87 (21982.67, 39751.97)	13.11 (9.59, 17.34)	−0.96 (−1.04, −0.89)
Southern Latin America	386.19 (234.74, 561.87)	1.99 (1.21, 2.90)	463.06 (294.91, 665.18)	2.37 (1.51, 3.41)	0.50 (0.37, 0.63)
Southern Sub-Saharan Africa	1161.77 (871.89, 1489.92)	4.39 (3.30, 5.63)	1300.54 (950.82, 1694.26)	4.16 (3.04, 5.42)	−0.19 (−0.26, −0.12)
Tropical Latin America	7887.79 (6158.79, 9818.30)	11.39 (8.89, 14.18)	20853.63 (16241.19, 26203.54)	31.32 (24.39, 39.35)	4.02 (3.53, 4.51)
Western Europe	3391.48 (2330.84, 4687.15)	3.45 (2.37, 4.77)	3542.39 (2531.41, 4774.27)	3.86 (2.76, 5.21)	0.50 (0.31, 0.69)
Western Sub-Saharan Africa	3183.69 (2285.77, 4180.97)	2.96 (2.13, 3.89)	8325.79 (5967.57, 10972.74)	3.10 (2.22, 4.09)	0.12 (0.06, 0.17)

UI, uncertainty interval; EAPC, estimated annual percentage change; SDI, Socio-Demographic Index.

**Table 2:** Number of DALYs and age-standardized DALYs rates per 100,000 population of acute glomerulonephritis among children and adolescents in 1990 and 2021 and temporal trends.

Characteristics	1990		2021		1990–2021
	DALYs cases no. (95%UI)	Rate/100,000	DALYs cases no. (95%UI)	Rate/100,000	EAPC no. (95%CI)
Overall	218260.11 (156771.09, 258757.07)	9.66 (6.94, 11.46)	59588.50 (32925.73, 79649.94)	2.26 (1.25, 3.02)	−4.33 (−4.46, −4.21)
Sex					
Male	122195.34 (63701.41, 149330.80)	10.56 (5.50, 12.90)	31895.69 (11267.90, 45739.76)	2.35 (0.83, 3.37)	−4.47 (−4.62, −4.32)
Female	96064.77 (75867.56, 126318.50)	8.72 (6.89, 11.47)	27692.82 (21015.50, 42593.59)	2.17 (1.65, 3.33)	−4.18 (−4.28, −4.07)
Socio-demographic factor					
High SDI	891.62 (728.47, 1125.07)	0.35 (0.29, 0.45)	477.49 (386.94, 578.42)	0.21 (0.17, 0.25)	−1.54 (−2.54, −0.52)
High-middle SDI	30854.46 (24002.05, 40115.38)	8.34 (6.48, 10.84)	3185.28 (2259.73, 4568.59)	1.05 (0.74, 1.51)	−6.48 (−6.57, −6.39)
Middle SDI	126286.86 (91929.53, 154070.80)	16.52 (12.02, 20.15)	23737.99 (14867.14, 32524.87)	3.17 (1.98, 4.34)	−4.91 (−5.08, −4.74)
Low-middle SDI	41517.23 (21857.76, 60406.35)	7.02 (3.70, 10.22)	13682.81 (7082.56, 18742.30)	1.79 (0.93, 2.45)	−4.17 (−4.23, −4.10)
Low SDI	18591.57 (7369.70, 29400.60)	6.65 (2.64, 10.52)	18458.84 (7775.30, 27347.07)	3.16 (1.33, 4.68)	−2.09 (−2.24, −1.95)
Region					
Andean Latin America	635.41 (420.02, 800.53)	3.35 (2.22, 4.22)	190.61 (130.35, 276.91)	0.81 (0.55, 1.17)	−4.93 (−5.90, −3.94)
Australasia	14.10 (10.17, 19.12)	0.22 (0.16, 0.30)	17.08 (10.85, 25.82)	0.23 (0.14, 0.34)	1.99 (−0.62, 4.66)
Caribbean	336.67 (243.76, 432.16)	2.23 (1.61, 2.86)	189.24 (130.30, 270.36)	1.24 (0.85, 1.77)	−1.32 (−1.97, −0.66)
Central Asia	2098.64 (1540.33, 2885.33)	6.65 (4.88, 9.14)	551.87 (398.52, 758.52)	1.59 (1.15, 2.19)	−4.78 (−5.28, −4.27)
Central Europe	497.77 (382.01, 636.71)	1.27 (0.97, 1.62)	189.25 (154.06, 235.00)	0.80 (0.65, 1.00)	−2.12 (−3.50, −0.73)
Central Latin America	975.87 (838.24, 1114.24)	1.18 (1.01, 1.35)	2759.00 (2160.87, 3547.70)	3.24 (2.53, 4.16)	4.94 (1.85, 8.13)
Central Sub-Saharan Africa	1730.26 (772.74, 2690.24)	5.58 (2.49, 8.68)	1413.17 (641.75, 2015.83)	1.92 (0.87, 2.74)	−2.91 (−3.23, −2.59)
East Asia	107291.24 (81698.89, 138226.25)	23.32 (17.76, 30.04)	9374.65 (6020.00, 13348.34)	2.72 (1.75, 3.87)	−6.47 (−6.64, −6.29)
Eastern Europe	3918.52 (3420.19, 4426.92)	5.82 (5.08, 6.58)	211.43 (179.32, 248.36)	0.46 (0.39, 0.54)	−8.92 (−9.54, −8.29)
Eastern Sub-Saharan Africa	8790.03 (2941.41, 14216.50)	7.93 (2.65, 12.82)	7393.83 (3000.53, 12484.09)	3.25 (1.32, 5.49)	−2.59 (−2.71, −2.47)
High-income Asia Pacific	70.75 (54.76, 92.22)	0.14 (0.11, 0.18)	29.48 (21.74, 40.97)	0.10 (0.07, 0.13)	−1.29 (−1.41, −1.17)
High-income North America	64.15 (56.65, 73.14)	0.08 (0.07, 0.09)	143.50 (118.10, 170.50)	0.16 (0.13, 0.19)	4.20 (1.54, 6.94)
North Africa and Middle East	5877.02 (4137.89, 7493.12)	3.32 (2.34, 4.24)	2956.48 (1610.02, 4264.53)	1.25 (0.68, 1.80)	−1.81 (−2.42, −1.20)
Oceania	2.42 (1.36, 4.12)	0.07 (0.04, 0.12)	3.50 (2.19, 5.44)	0.05 (0.03, 0.09)	−0.86 (−0.99, −0.73)
South Asia	6007.65 (3392.46, 7979.75)	1.11 (0.63, 1.47)	1948.39 (1329.81, 2774.52)	0.29 (0.19, 0.41)	−4.45 (−4.71, −4.18)
Southeast Asia	61518.58 (29866.89, 93375.33)	27.98 (13.58, 42.46)	19619.77 (10348.21, 27992.10)	8.56 (4.51, 12.21)	−3.51 (−3.62, −3.40)
Southern Latin America	47.09 (33.11, 64.93)	0.24 (0.17, 0.34)	45.70 (34.46, 61.10)	0.23 (0.18, 0.31)	0.19 (−1.49, 1.91)
Southern Sub-Saharan Africa	940.43 (663.43, 1332.91)	3.55 (2.51, 5.04)	878.65 (575.06, 1255.65)	2.81 (1.84, 4.02)	−0.14 (−0.44, 0.16)
Tropical Latin America	8722.96 (7186.04, 10347.20)	12.59 (10.38, 14.94)	1010.31 (788.92, 1235.92)	1.52 (1.18, 1.86)	−6.32 (−6.78, −5.86)
Western Europe	109.17 (93.75, 125.80)	0.11 (0.10, 0.13)	84.56 (70.79, 98.01)	0.09 (0.08, 0.11)	−0.07 (−1.71, 1.60)
Western Sub-Saharan Africa	8611.38 (2800.93, 14367.30)	8.01 (2.61, 13.37)	10578.03 (4264.27, 15406.16)	3.94 (1.59, 5.74)	−1.76 (−1.98, −1.55)

DALYs, Disability-Adjusted Life Years; EAPC, estimated annual percentage change; UI, uncertainty interval; CI, confidence interval.

### AGN in children and adolescents among different countries and territories

**Figure 1:**
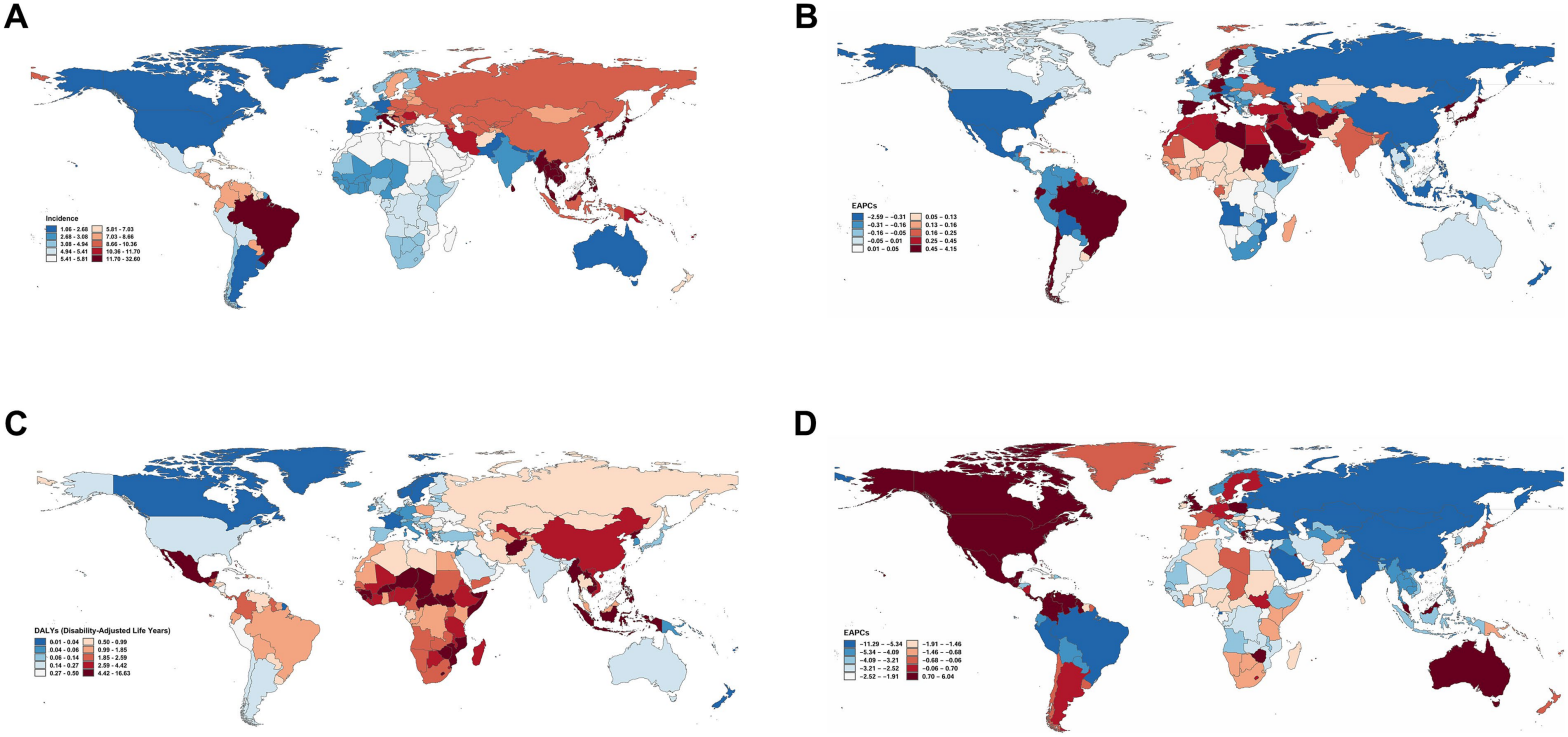
Global maps of incidence and DALYs of acute glomerulonephritis among children and adolescents in 204 countries and territories, 1990–2021. **(A)** Incidence rate in 2021 (per 100,000 population). **(B)** EAPC in incidence rate from 1990 to 2021. **(C)** The age-standardized DALYs rate in 2021 (per 100,000 population). **(D)** EAPC in DALYs rate from 1990 to 2021 (with 95% CI). DALYs, disability-adjusted life years; EAPC, estimated annual percentage change. Data source: Global Burden of Disease Study 2021.

**Figure 2:**
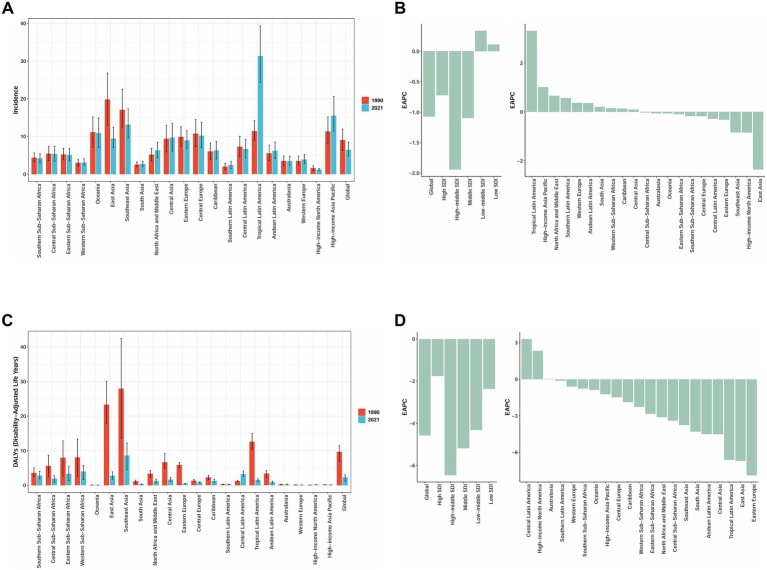
Change in Incidence and DALYs for acute glomerulonephritis among children and adolescents in 21 GBD Regions, 1990–2021. **(A)** Incidence rate (per 100,000 population, with 95%UI). **(B)** EAPC in incidence rate (with 95% CI). **(C)** The age-standardized DALYs rate (per 100,000 population, with 95%UI). **(D)** EAPC in DALYs rate (with 95% CI). DALYs, disability-adjusted life years; GBD, Global Burden of Disease, EAPC: estimated annual percentage change. Data source: Global Burden of Disease Study 2021.

According to the GBD 2021 report, a total of 204 countries and territories worldwide have documented cases of AGN in children and adolescents, which contributed to AGN-associated DALYs in these regions. In 2021, China reported the largest number of cases, totaling 29,608 (95% UI: 21,974–39,040), accounting for over 17% of global pediatric incident cases. It was followed by Brazil with 20,631 cases (95% UI: 16,066–25,894) and India with 13,514 cases (95% UI: 10,328–17,252) (Supplementary Table S2). There was substantial international variation in the incidence rate of AGN among children and adolescents in 2021. The highest incidence rates were observed in Brazil (32.28 per 100,000), the Democratic People’s Republic of Korea (29.27 per 100,000), and Taiwan (Province of China) (25.79 per 100,000), all notably surpassing the global mean incidence rate of 7.20 per 100,000, with 81 countries exhibiting rates above this average. In contrast, Greece reported the lowest incidence rate at 1.07 per 100,000 (Figure 1A). An assessment of temporal trends revealed pronounced divergence: out of the 204 countries and territories, 128 showed an increasing incidence trend, with Brazil demonstrating the most significant increase (EAPC = 4.11, 95% CI: 3.61 to 4.62), while China experienced the most marked decrease (EAPC = −2.56, 95% CI: −2.81 to −2.31) (Figure 1B). From 1990 to 2021, different regions exhibited significant variations. High-income regions, such as North America and Western Europe, demonstrated relatively stable incidence rates, while Tropical Latin America and East Asia experienced more pronounced fluctuations (Figure 2A). The East Asian region, particularly China, showed a significant downward trend, whereas Tropical Latin America displayed a continuous upward trend (Figure 2B).

In 2021, the global distribution of DALYs for childhood and adolescent AGN demonstrated substantial geographical heterogeneity. The highest age-standardized DALY rates were observed in the Lao People’s Democratic Republic (16.47 per 100,000; 95% UI: 7.26–29.56), Timor-Leste (16.08 per 100,000; 95% UI: 7.91–26.16), and Myanmar (13.98 per 100,000; 95% UI: 5.40–22.73), significantly exceeding the global average (Figure 1C). In terms of absolute DALY numbers, China (9,069; 95% UI: 5,866–13,070), Indonesia (7,493; 95% UI: 3,566–12,383), and the Philippines (6,157; 95% UI: 4,400–8,295) bore the heaviest burden (Supplementary Table S3), collectively accounting for a substantial proportion of the global total. From 1990 to 2021, the most rapid increases in age-standardized DALY rates were observed in Mexico (EAPC = 5.98, 95% CI: 2.43 to 9.65), while Georgia (EAPC = −11.18, 95% CI: −12.30 to −10.05) experienced the most pronounced declines (Figure 1D). Middle and high SDI regions demonstrated more significant trends in DALY changes (Figure 2C), with Central Latin America and high-income North America showing upward trends. Most regions experienced a decline in DALYs, with Eastern Europe exhibiting the most notable downward trend in DALYs (Figure 2D). To better visualize the temporal trends in the AGN burden, we have also generated incidence and DALYs time-series analyses for key countries, including China, Brazil, India, Laos, Timor-Leste, Mexico, and Georgia. The corresponding figures and numerical data are provided in Supplementary Figures S1, S2; Supplementary Data sheet S1.

### AGN in children and adolescents among different SDI regions

**Figure 3:**
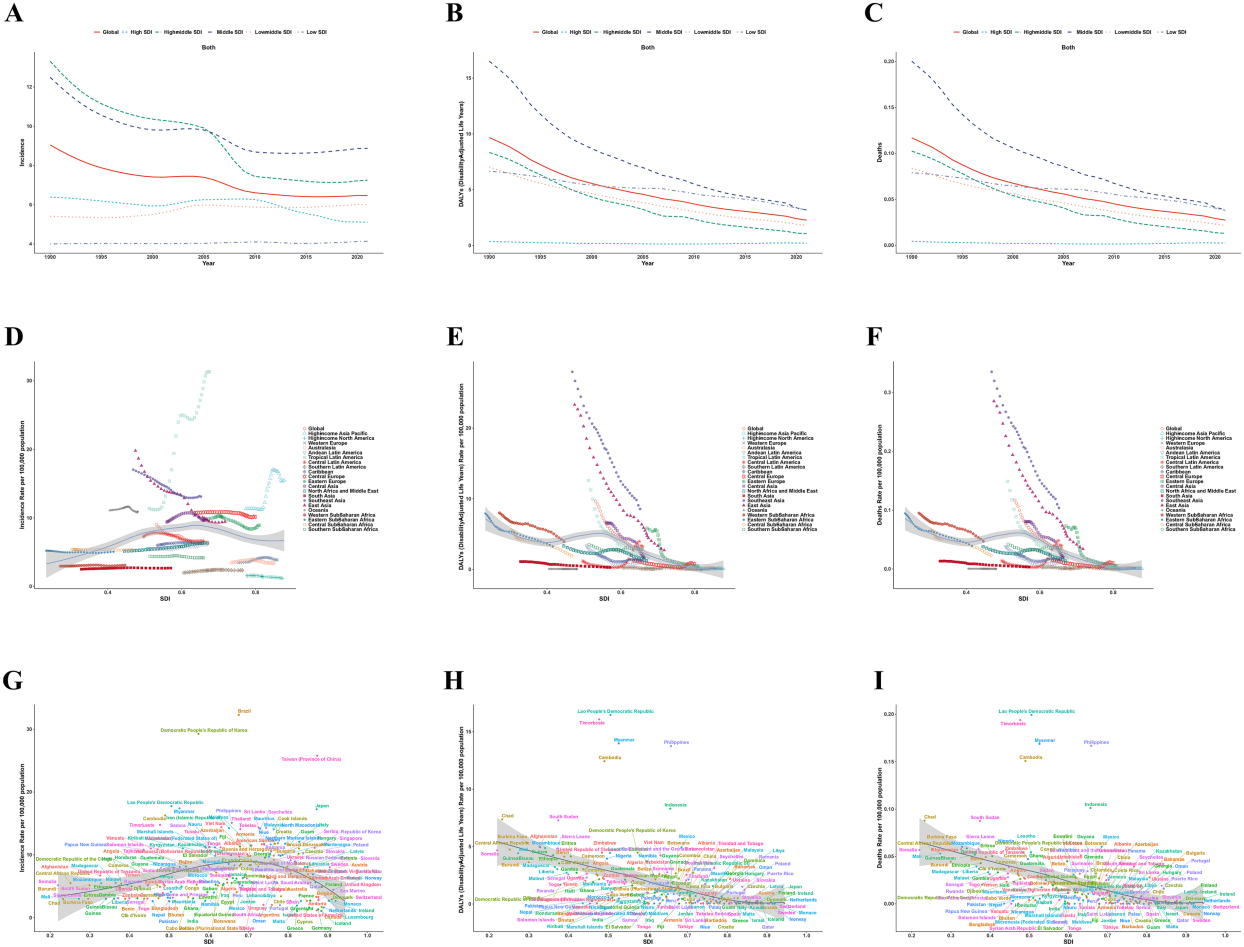
Burden of acute glomerulonephritis among children and adolescents by SDI, 1990–2021. **(A–C)** represent the relationship between the disease burden and times across globally and 5 SDI regions. **(A)** Incidence rate. **(B)** DALYs rate. **(C)** Deaths rate. D-F represent the relationship between the disease burden and SDI across 21 GBD regions. **(D)** Incidence rate. **(E)** DALYs rate. **(F)** Deaths rate. **(G–I)** represent the relationship between the disease burden and SDI across 204 countries and territories. **(G)** Incidence rate. **(H)** DALYs rate. **(I)** Deaths rate. DALYs, disability-adjusted life years; SDI, Socio-Demographic Index; GBD, Global Burden of Disease. Data source: Global Burden of Disease Study 2021.

The stratified analysis based on the SDI revealed a distinct epidemiological gradient in the burden of AGN among children and adolescents (Figure 3). Incidence rates in high-middle and middle SDI regions consistently exceeded the global average throughout the observation period. High-middle SDI regions initially reported the highest rates at 13.31 (95% UI: 8.50–18.20) in 1990, but showed a significant decline after 2006 to 7.25 (95% UI: 5.18–9.69), stabilizing thereafter in 2015. High SDI regions maintained consistently low levels, with a continuous decline from 6.39 (95% UI: 4.42–8.61) to 5.10 (95% UI: 3.69–6.81), while low-middle SDI regions experienced a gradual increase from 5.42 (95% UI: 4.03–7.05) to 6.01 (95% UI: 4.48–7.76). In contrast, low SDI regions remained stable with minimal fluctuations (4.00–4.14). Both DALYs and mortality rates showed a continuous downward trend, albeit with significant variations across different SDI regions. High SDI regions consistently maintained the lowest disease burden, with a DALY rate in 2021 of only 0.21 (95% UI: 0.17–0.25), representing a 42.2% decrease from 1990. Middle SDI regions experienced the most significant reduction in DALYs, decreasing from 16.52 (95% UI: 12.02–20.15) in 1990 to 3.17 (95% UI: 1.98–4.34) in 2021, a decline of 80.8%. Although low SDI regions also exhibited a downward trend in DALYs (3.16 in 2021, 95% UI: 1.33–4.68), they continued to bear the heaviest disease burden. Middle SDI regions had the highest mortality rate but demonstrated a significant reduction (Figures 3A–C).

There was no statistically significant association between AGN incidence rates and the SDI across 21 regions and globally for children and adolescents (*r* = 0.0228, 95% CI: −0.0586 to 0.1069, *p* = 0.5452). Low SDI regions, such as sub-Saharan Africa and South Asia, displayed significant fluctuations in incidence rates. In contrast, SDI and DALYs demonstrated a significant negative correlation (*r* = −0.5444, 95% CI: −0.6061 to −0.4774, *p* < 0.001). Regional differences were notable: East Asia and Southeast Asia exhibited the highest DALY rates in the early 1990s, while high-income North America had the lowest rates. The SDI gradient revealed that low SDI regions, such as sub-Saharan Africa, consistently maintained higher DALY rates than high SDI regions, such as Western Europe, with the DALY rate in East Sub-Saharan Africa being 20 times higher than that in high-income North America in 2021 (Figures 3D–F). Spearman correlation analysis of AGN incidence and SDI across 204 countries and territories indicated that AGN incidence had an extremely weak positive correlation with SDI (r = 0.0835, 95% CI: −0.0878 to 0.2318); however, this correlation was not statistically significant (*p* = 0.235). Conversely, DALYs demonstrated a strong negative correlation with SDI (r = −0.6887, 95% CI: −0.7681 to −0.5986, *p* < 0.001). Countries with lower SDIs (e.g., sub-Saharan Africa, Southeast Asia) had significantly higher AGN DALY rates (e.g., Cambodia, Myanmar, Philippines > 10/100,000), whereas high-SDI nations (e.g., Western Europe, North America) reported rates below 1/100,000. This analysis revealed that high DALY rates and low SDI clustered in Southeast Asian and sub-Saharan African countries, while low DALY rates and high SDI clustered in high-income countries (Figures 3G–I).

### Age and sex differences

**Figure 4:**
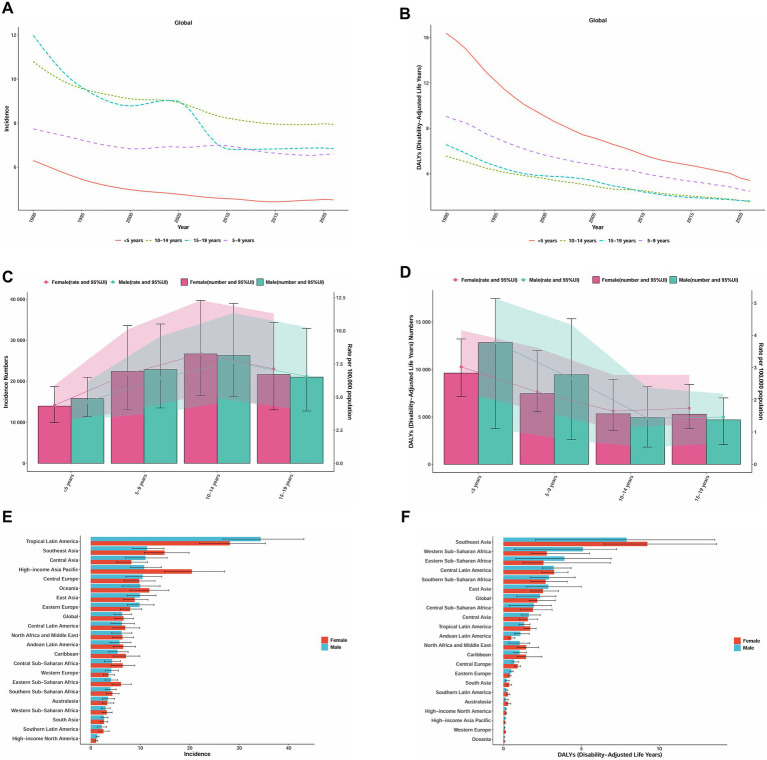
Trends in incidence rate, DALYs for acute glomerulonephritis in children and adolescents by age and sex, 1990–2021. **(A)** Incidence rate by age (per 100,000 population, with 95%UI). **(B)** DALYs rate by age (per 100,000 population, with 95%UI). **(C)** Incidence number and rate by sex in 2021. **(D)** DALYs number and rate by sex in 2021. **(E)** Incidence rate in 21 GBD Regions by sex in 2021 (per 100,000 population). **(F)** DALYs rate in 21 GBD Regions by sex in 2021(per 100,000 population). DALYs, disability-adjusted life years; GBD, Global Burden of Disease. Data source: Global Burden of Disease Study 2021.

Analysis of the long-term trends in the burden of AGN by age groups from 1990 to 2021 revealed distinct patterns for both incidence and DALYs (Figures 4A,B). All age groups demonstrated declining trends in incidence rates, although with varying trajectories. The 10–14 year age group consistently maintained the highest incidence rates, while the 15–19 year age group exhibited the most dramatic changes, converging with the 5–9 year age group by 2010. Notably, children under 5 years consistently showed the lowest incidence rates throughout the entire period. In contrast, DALY rate trends exhibited a different pattern, with the under 5 year age group bearing the highest burden across the entire period, despite showing the most substantial decline. The 5–9 year age group maintained the second-highest DALY burden, while the 10–14 year and 15–19 year age groups exhibited similar trajectories. These contrasting patterns between incidence and DALY trends highlight that while disease occurrence remains highest in older children and adolescents, the severity and impact of the disease are disproportionately concentrated in the youngest children.

Our analysis of age and sex-specific patterns in the global burden of AGN in 2021 revealed several notable findings (Figures 4C,D). Peak incidence occurred in the 10–14 year age group (males: 26,262 cases, 95% UI: 16,264–38,967; females: 26,671 cases, 95% UI: 16,537–39,696), rather than following a simple age-related decline. Incidence rates were higher in females across most age groups, except for children under 5 years. The DALY burden exhibited a different distribution, with the highest burden concentrated in children under 5 years, particularly among males (12,829, 95% UI: 3,770–17,470), which is approximately 1.3 times higher than their female counterparts (9,615, 95% UI: 7,160–13,215). We observed an age-related shift in sex differences: males bore a greater DALY burden in younger age groups (under 5 years: males 3.77 vs. females 3.02 per 100,000), while females had a higher burden in adolescents (15–19 years: females 5,283 vs. males 4,694 DALYs).

**Figure 5:**
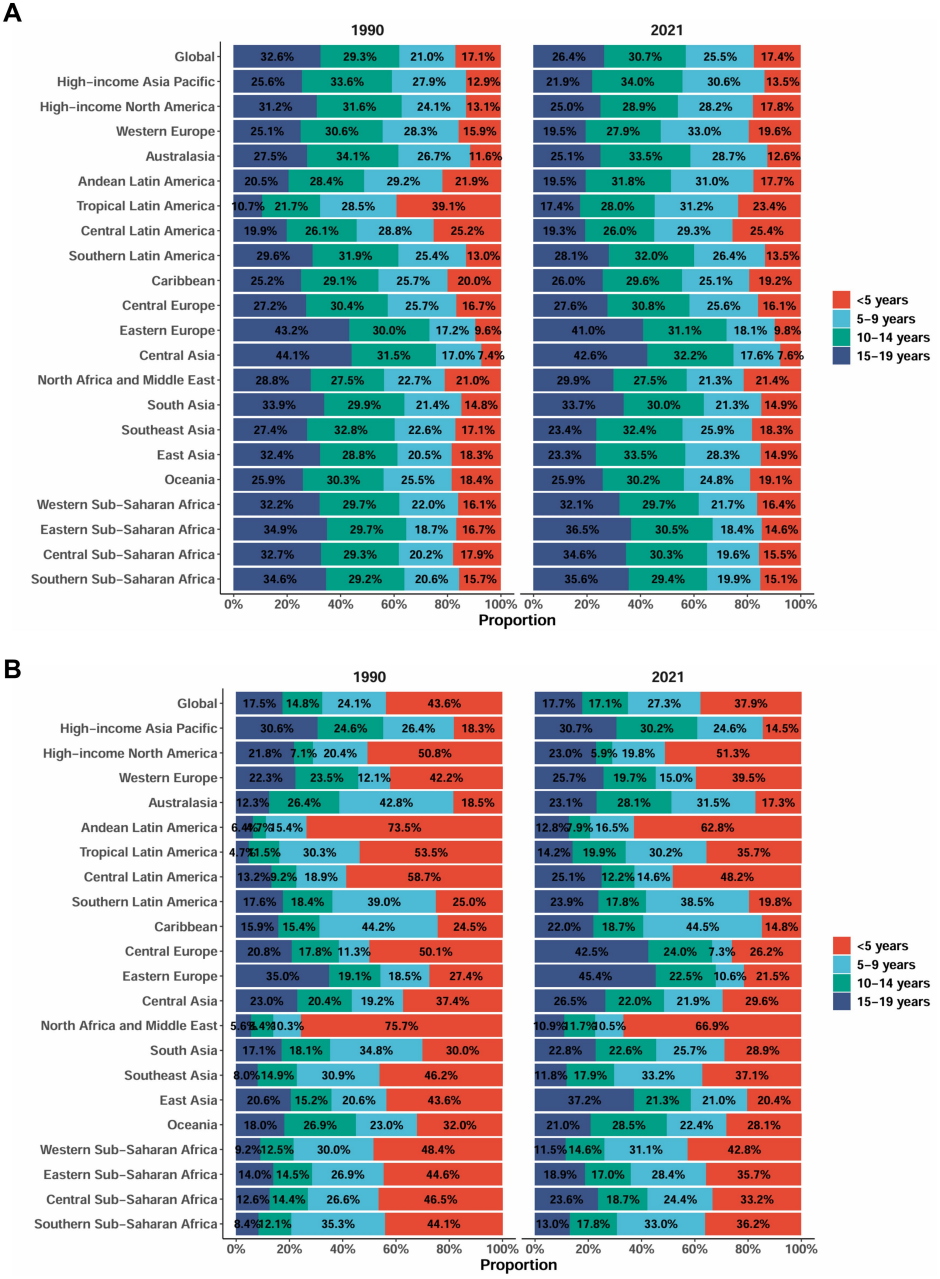
Age-specific percentages of acute glomerulonephritis among children and adolescents in incidence and DALYs in 2021. **(A)** Incidence rate (per 100,000 population). **(B)** DALYs rate (per 100,000 population). DALYs, disability-adjusted life years. Data source: Global Burden of Disease Study 2021.

Regional and sex differences in the burden of AGN in 2021 revealed substantial disparities. The highest incidence rates were observed in Tropical Latin America, followed by high-income Asia Pacific and Southeast Asia. Notably, the pattern of sex differences varied by region: females exhibited significantly higher incidence rates in high-income Asia Pacific (female-to-male ratio: 1.89) and the Caribbean (1.31), while males predominated in Central Asia (male-to-female ratio: 1.35) and Eastern Europe (1.24). The DALY burden demonstrated a different geographical pattern, with Southeast Asia exhibiting the highest rates, followed by Western Sub-Saharan Africa and Eastern Sub-Saharan Africa (Figures 4E,F). The age structure of AGN incidence by region and globally, from 1990 to 2021, reveals a notable decline in the proportion of cases among children under 5 years (32.6% in 1990 to 26.4% in 2021), while the share for ages 10–14 years increased (21.0 to 25.5%) globally. This pattern is consistent across most regions. Some regions, such as Central Asia and Eastern Europe, show particularly pronounced increases in the older age groups (≥ 10 years), while the share of the ≤ 5 years group decreased or remained stable. Globally and in most regions, the proportion of DALYs attributable to the youngest age group (< 5 years) remained relatively high from 1990 (43.6%) to 2021 (37.9%), although a gentle decline is evident. Nevertheless, in several regions such as Eastern Europe, the DALY burden in the < 5 years group increased, while the proportions in the 10–14 years and 15–19 years groups generally declined or remained stable. Notably, in some high-burden regions (Central Asia, North Africa, and the Middle East), the < 5 years category consistently dominated DALY composition (Figures 5A,B).

### Cross country inequality analysis

**Figure 6:**
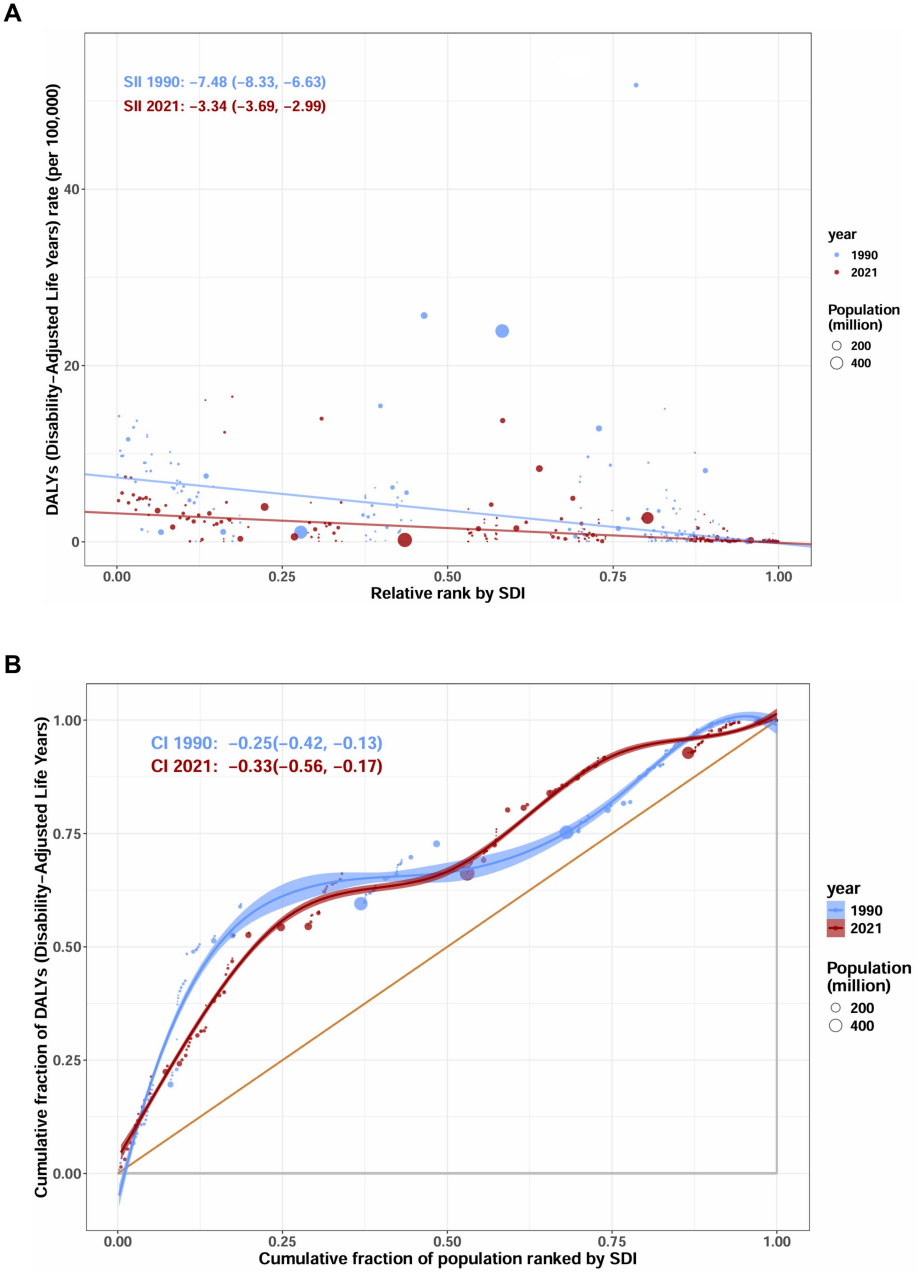
SDI-related health inequality assessment of DALYs for acute glomerulonephritis in children and adolescents. **(A)** Slope index of inequality (SII) for DALYs rate. **(B)** Concentration index (with 95%CI) of inequality for DALYs rate. DALYs, disability-adjusted life years. Data source: Global Burden of Disease Study 2021.

In terms of cross-country inequality, our analysis revealed substantial absolute and relative disparities in the burden of AGN associated with SDI levels. Generally, countries and regions with lower SDI disproportionately bear a higher disease burden. The concentration index for DALY rates was −0.25 (95% CI: −0.42 to −0.13) in 1990 and decreased further to −0.33 (95% CI: −0.56 to −0.17) by 2021. The increased absolute concentration index of inequality magnitude suggests a slight increase in relative inequality (Figure 6B). Regarding absolute inequality, the attenuation of the SII magnitude from −7.48 (95% CI: −8.33 to −6.63) in 1990 to −3.34 (95% CI: −3.69 to −2.99) in 2021 is noteworthy (Figure 6A). These results indicate that although absolute inequality in AGN burden between countries of different SDI levels has decreased over time, relative inequality remains pronounced and even shows a modest upward trend. Overall, disadvantaged regions continue to bear a disproportionate AGN disease burden, highlighting the urgent need for targeted interventions and equitable healthcare strategies.

### Prediction of AGN incidence and DALYs rate to 2050

**Figure 7:**
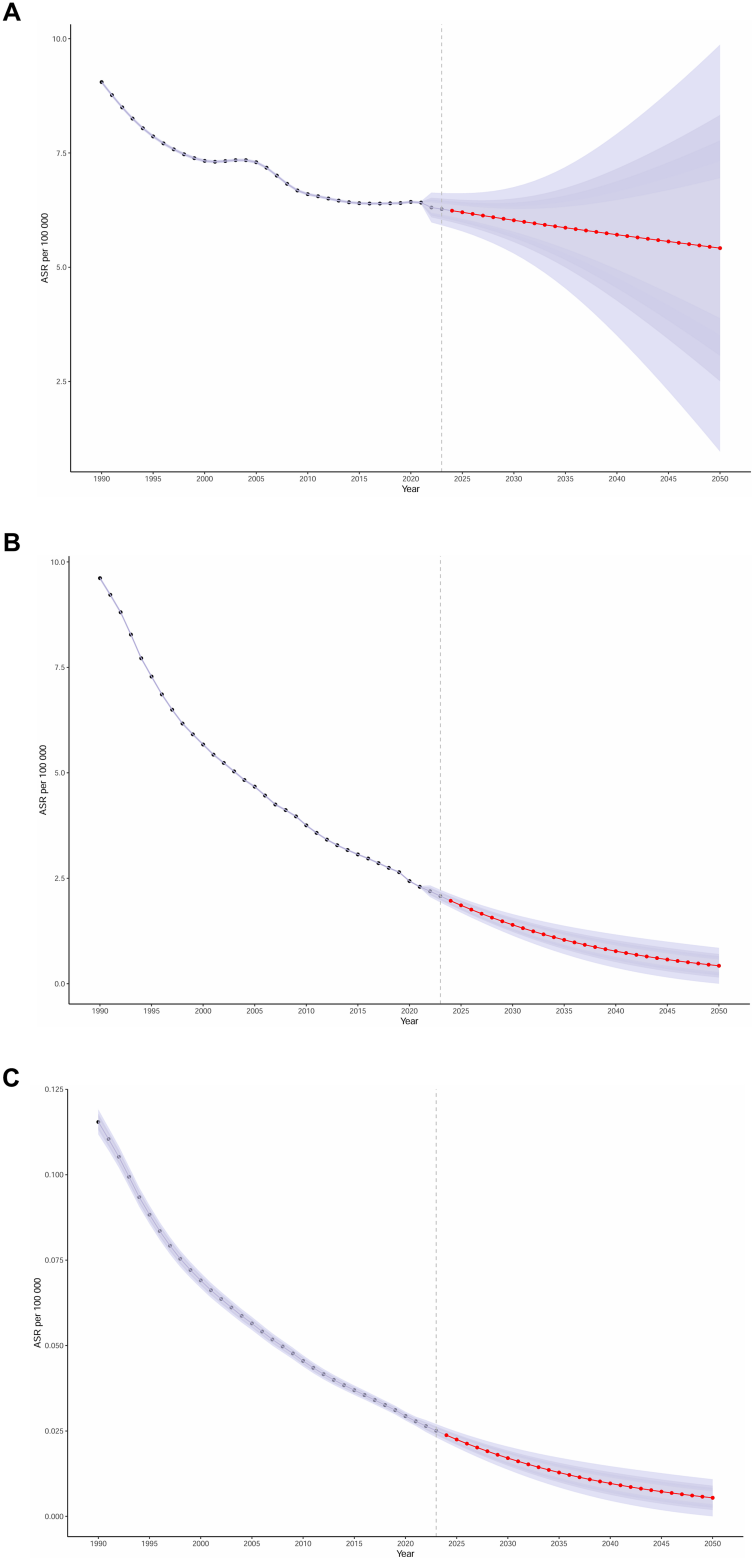
Projection of acute glomerulonephritis in children and adolescents in incidence, DALYs, deaths trend to 2050. **(A)** Incidence rate (per 100,000 population, with 95%UI). **(B)** DALYs rate (per 100,000 population, with 95%UI). **(C)** Death rate (per 100,000 population, with 95%UI). DALYs, disability-adjusted life years. Data source: Global Burden of Disease Study 2021.

Based on the GBD database and projections using the BAPC model, we analyzed and forecasted the long-term trends of global ASIRs, mortality, and DALY rates for AGN from 1990 to 2050. From 1990 to 2019, the ASR exhibited a steady annual decline of 1.2%. Our model predicts that this declining trend will continue, albeit at a slower rate of 0.57% annually from 2020 to 2050, with the ASR projected to decrease from 6.43 per 100,000 in 2020 to 5.42 per 100,000 by 2050, representing a total reduction of 15.72%. Mortality rates are also expected to decrease continuously. The DALY rate is projected to reach 1.40 per 100,000 (95% CI: 1.14–1.65) in 2030, with a further decline that slows after 2040, ultimately reaching 0.77 per 100,000 (95% CI: 0.64–0.91) in 2050. Overall, our projections suggest a continued but decelerated improvement in the global burden of AGN through 2050 (Figure 7; Supplementary Tables S4, S5).

We then assessed model adequacy using posterior predictive checks, comparing observed under-20 aggregated rates with the model’s posterior predictions for 1990–2021. Observed rates closely tracked predictions, with RMSE = 0.04, MAE = 0.04, MAPE = 0.57, and 95% predictive coverage = 40.6% (Supplementary Table S6; Supplementary Figure S3).

### Frontier analysis

**Figure 8:**
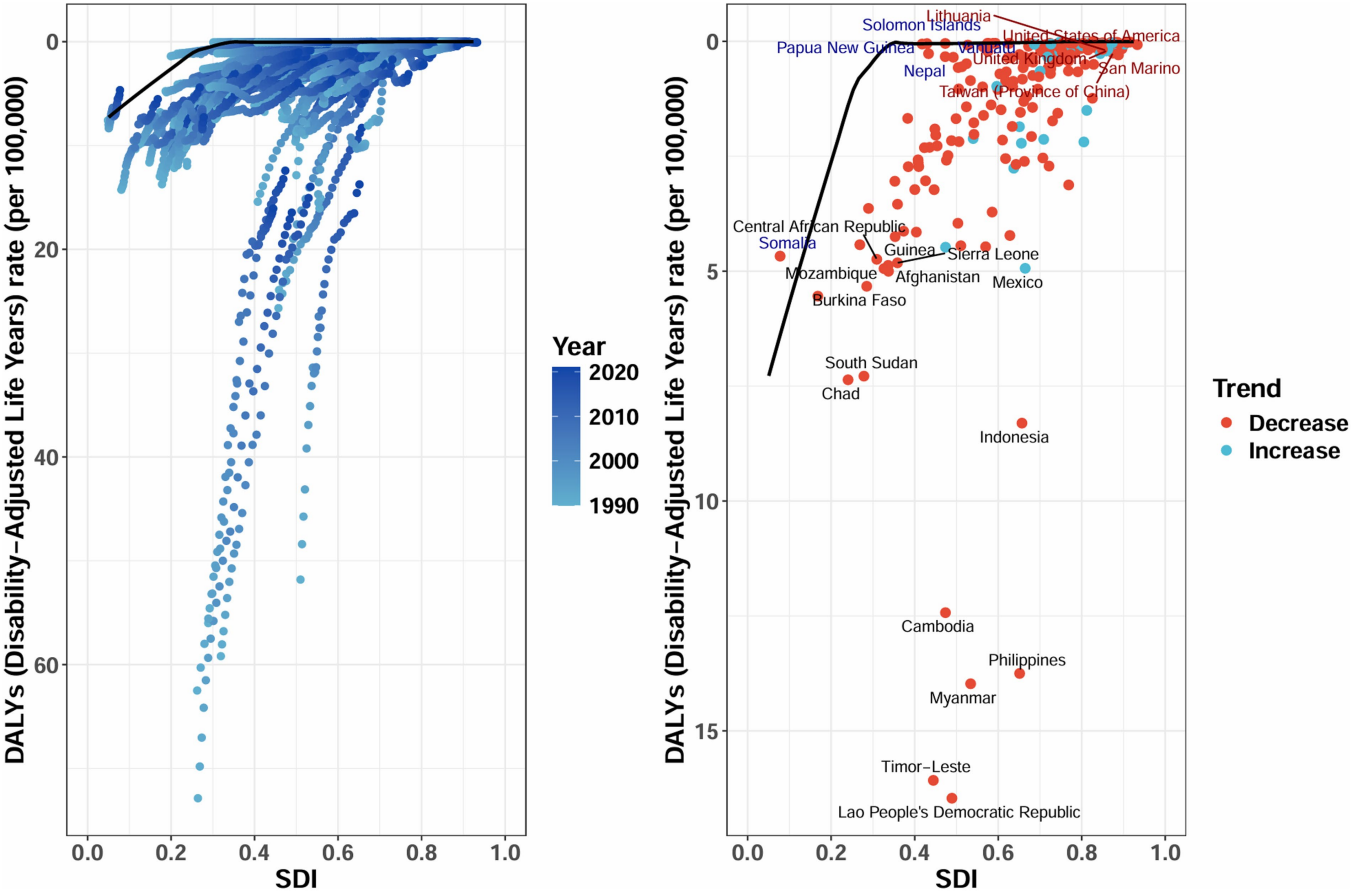
Frontier analysis exploring the relationship between SDI and age-standardized DALYs rate for acute glomerulonephritis in children and adolescents across 204 countries and territories. Border is delimited in solid black; Countries and territories are represented by dots. The DALYs rate is presented per 100,000 population. SDI, Socio-Demographic Index; DALYs, disability-adjusted life years. Data source: Global Burden of Disease Study 2021.

We employed age-standardized DALY rates to investigate the relationship between the burden of AGN and the SDI globally from 1990 to 2021. This analysis leveraged frontier techniques to evaluate unrealized health gains at both the country and regional levels (Figure 8). The largest efficiency gaps were identified in Southeast Asian nations, with Laos (Lao PDR) (efficiency difference: 16.42) and Timor-Leste (16.03) experiencing DALY rates more than 16-fold above theoretical optimal values, despite their intermediate SDI status (0.49 and 0.44, respectively). The Philippines (SDI = 0.65) demonstrates paradoxically high DALYs (13.72), despite its moderate development. Among high-SDI nations (SDI > 0.75), countries like the United Kingdom and the United States maintain efficiency gaps below 0.3, with absolute DALY rates at exceptionally low levels (0.17–0.29), demonstrating diminishing returns on additional investments. Over the period from 1990 to 2021, nearly all countries showed a downward trajectory in DALY rates as SDI increased; however, the pace and magnitude of this decline varied considerably. High-performing countries converged toward the frontier and maintained low DALY rates throughout this period, while several low- and middle-SDI countries achieved only modest reductions and remained substantially above the benchmark.

## Discussion

To our knowledge, this is the first comprehensive systematic study examining the global, regional, and national disparities of AGN in children and adolescents. Additionally, this research provides a thorough assessment based on trend analysis, inequality evaluation, frontier analysis, and predictive modeling. Despite the high incidence of AGN in children, there exists a lack of comprehensive global disease burden reporting for this population. Our study reveals a significant and detailed global disease burden, estimating 170,584.89 new cases in 2021, with an ASIR of 6.47 per 100,000 population. Both the absolute number of cases and the ASIR showed a declining trend from 1990 to 2021, revealing a true reduction in disease risk. From 1990 to 2021, the AGN burden showed a continuous declining trend, with an EAPC of −1.03% for incidence and −4.33% for DALYs. Notably, while global incidence is declining, significant regional and socioeconomic disparities persist. In 2021, China had the highest number of cases and experienced the most substantial decline in incidence (EAPC = −2.56), whereas Brazil demonstrated the most significant increase (EAPC = 4.11), with 128 out of 204 countries and territories showing an upward trend. Middle SDI regions had the highest incidence rate, at 1.74 times that of high SDI regions, while lower-middle and low SDI regions exhibited counterintuitive increases in incidence (EAPC of 0.42 and 0.07%, respectively), highlighting the complex epidemiological landscape of AGN. The study’s findings emphasize the critical importance of understanding the dynamic burden of AGN in children and adolescents, with the disease burden shifting towards resource-limited regions. These insights are crucial for developing targeted public health interventions and resource allocation strategies.

Our study reveals that in 2021, China accounted for over 17% of the global incidence of AGN, followed by Brazil and India. The burden of AGN is primarily borne by developing countries, which is consistent with previous research (13). Interestingly, the highest AGN incidence in children and adolescents was observed in tropical Latin America, coupled with a relatively moderate DALY rate, suggesting effective disease management despite the high incidence. Conversely, regions like sub-Saharan West Africa exhibited disproportionately high DALY rates compared to incidence, indicating potentially more severe disease manifestations or limited medical accessibility. While our study demonstrates a declining trend in the age-standardized DALY rates for AGN, it is crucial to acknowledge that the DALY model primarily captures the acute disease burden. It may systematically underestimate the most significant long-term health risk of AGN: the progression to chronic kidney disease (CKD). A substantial body of evidence indicates that a subset of children who clinically ‘recover’ from AGN continue to harbor subclinical renal injury, manifesting as persistent microalbuminuria, hypertension, or a slow decline in the glomerular filtration rate (5, 22, 23). In low- and middle-income countries, post-infectious glomerulonephritis (PIGN) remains a common cause of severe acute kidney injury, often requiring dialysis and pediatric intensive care unit admission (3). These sequelae, particularly persistent proteinuria and hypertension, constitute the primary pathogenic drivers for the development and progression of future CKD. Consequently, the DALYs reported in our study do not fully account for the substantial future burden of CKD attributable to AGN, which requires decades of management by nephrologists (8). Our findings, especially the persistently high incidence of AGN in low and middle SDI regions, foreshadow a more insidious and substantial future burden of CKD. This insight is critical for public health policymakers, interventions targeting AGN must extend beyond reducing acute incidence and mortality. It is imperative to establish robust long-term follow-up systems for AGN survivors, incorporating regular monitoring of blood pressure and urinary protein, to genuinely curb the progression to CKD and alleviate the long-term burden on healthcare systems.

The DALY burden of AGN in children and adolescents demonstrates significant geographical heterogeneity globally. Countries with the highest age-standardized DALY rates include Laos, Timor-Leste, and Myanmar. From 1990 to 2021, Mexico experienced the largest absolute increase in DALY rate, while Georgia showed the most substantial decline. These findings reveal notable regional burden disparities and dynamic epidemiological transitions, emphasizing the need to prioritize high-burden countries and those with rapidly changing trends for future public health interventions. The decline in age-standardized rates (ASR) from 1990 to 2019 may be attributed to improved global health conditions, enhanced prevention and control of streptococcal infections, and advances in medical technology (3, 24). Over the past 40 years, the significant reduction in PSAGN incidence in the United States is primarily credited to better sanitation conditions and/or a decreased prevalence of M-type kidney pathogen subtypes associated with skin infections, which has led to the near-eradication of streptococcal pyoderma (13). Guo et al. (6) analyzed the global AGN burden from 1990 to 2019 based on the GBD study, finding an overall declining incidence trend, with particularly high disease burden observed among children and older adults populations. Teng et al. (25) noted that AGN mortality trends varied globally, with high SDI countries and regions showing an increasing mortality rate, while other countries demonstrated a declining trend. The interpretation of AGN-associated mortality requires consideration of potential confounding factors, with hypertension being particularly relevant. Severe hypertension is a well-recognized complication of AGN that can contribute directly to mortality through hypertensive encephalopathy, heart failure, or cerebrovascular events (24, 26, 27). While the GBD methodology aims to attribute mortality to the underlying cause (AGN), the possibility remains that the presence and severity of hypertension or other complications (such as acute kidney injury) might act as important mediators or confounders in the pathway from AGN to death (26). This could potentially lead to either over-estimation or under-estimation of the true mortality burden attributable specifically to AGN, particularly in settings with limited diagnostic capabilities. The disproportionately high burden of AGN in low-SDI regions is predominantly driven by Acute Post-Infectious Glomerulonephritis (APIGN), with post-streptococcal glomerulonephritis (PSAGN) representing the most prevalent form. This epidemiological pattern is strongly supported by existing evidence: post-infectious glomerulonephritis remains the primary cause of AGN in children worldwide, typically following group A streptococcal infections, with streptococcal pharyngitis being the predominant trigger for PSAGN in children (13, 22, 28). The compelling case of Indigenous children in central Australia, who experience the highest reported incidence of PSAGN globally (1), exemplifies how specific population groups facing socioeconomic challenges bear the heaviest disease burden. Critically, the incidence burden of AGN is predominantly concentrated in pediatric populations, with global estimates indicating approximately 404,000 annual cases of PSAGN occurring in children out of a total of 472,000 cases (13). This concentration in children, combined with the well-documented association between resource-limited settings, characterized by overcrowding, poor sanitation, and limited healthcare access, and increased risk of streptococcal infections (2, 3, 8), provides compelling evidence that the heavy AGN burden in low-SDI settings is indeed predominantly APIGN. This etiological clarity justifies prioritizing targeted interventions in these regions, focusing on primary prevention through improved hygiene and sanitation, alongside early detection and antibiotic treatment of streptococcal infections to prevent APIGN development (26).

Among children and adolescents of various ages, the 10–14 age group exhibits the highest incidence of AGN but presents lower DALYs, while children under 5 years show the lowest incidence yet bear the most severe burden of DALYs. The patterns of AGN incidence and DALY rates across age groups reveal a significant contrast, with disease severity and impact disproportionately concentrated in the youngest children (<5 years), despite a higher occurrence in older children (10–14 years). This disparity may arise from several factors. First, lower rates of streptococcal pharyngitis and immature immune (or antibody) responses in younger age groups could lead to more severe disease manifestations and complications, including acute kidney injury and systemic inflammation (13, 22). Second, young children are more vulnerable to volume loss, electrolyte imbalances, and drug toxicity, which may complicate treatment (29). These findings have important research and clinical implications; they emphasize the necessity of heightened vigilance, early intervention, and more intensive management for young children with AGN. From a public health perspective, the substantial reduction in DALY rates for children under 5 years, decreasing from 16.4 to 3.5 per 100,000 from 1990 to 2021, represents a major success, likely due to improved medical accessibility, earlier diagnosis, and advancements in pediatric nephrology care, including widespread antibiotic use and enhanced socioeconomic and nutritional conditions (3, 22, 24, 27, 30). However, the persistent disparities among age groups highlight the ongoing need to develop age-appropriate prevention strategies and clinical protocols for this vulnerable population. Future research should explore the biological mechanisms underlying these age-specific differences to inform more targeted therapeutic approaches.

Our study reveals age-dependent gender differences in AGN among children and adolescents. Males bear a higher DALYs burden in younger age groups (<5 years), while females experience a greater burden during adolescence (15–19 years). These dynamic variations may be linked to the potential modulatory effects of immune systems and hormones throughout the developmental process (31, 32). Patterns of gender differences exhibit regional variations. In high-income Asia-Pacific and Caribbean regions, female incidence rates are significantly higher than those of males, whereas Central and Eastern European regions demonstrate the opposite trend. Such disparities in geographical distribution suggest a substantial influence of genetic backgrounds, environmental factors, and socioeconomic conditions on AGN incidence patterns (22).

Based on the BAPC model, our research predicts that the Age-Standardized Rate (ASR) decline will continue until 2050, although the improvement rate will slow to 0.57% annually. Regarding DALYs, the rate of decline is expected to decelerate after 2040, reaching 0.77 per 100,000 by 2050. The period effect reflects the impact of medical technological advancements, while the cohort effect highlights the need to focus on long-term health risks for specific birth cohorts. These findings emphasize the ongoing importance of maintaining global measures for the prevention and management of AGN, despite existing progress, and underscore the necessity of remaining vigilant about potential bottlenecks in control efficiency. The predictive model illustrates the nuanced trajectory of AGN epidemiology, indicating that while significant improvements have been made, sustained and adaptive public health strategies are essential for ongoing disease management and reduction.

The analysis of AGN inequality reveals an intensification of health disparities, with the concentration index of inequality for DALYs increasing in absolute value from −0.25 to −0.33. This indicates a deepening concentration of AGN disease burden among low-income populations, potentially linked to widening socioeconomic gaps, unequal distribution of medical resources, or insufficient preventive measures (33–36). For example, central Australia has taken urgent action to mitigate the related disease burden by improving housing conditions and alleviating population overcrowding in towns and communities (1). This analysis confirms the strong correlation between kidney disease burden and socioeconomic factors, emphasizing the need to strengthen early screening, public health education, and medical accessibility in low-income regions to reduce health inequalities. The SII remained negative in 2021, underscoring the necessity for targeted policies to enhance primary healthcare coverage and health education for low-income groups in the prevention and control of kidney disease (37).

Using the frontier analysis method, we quantified the efficiency gaps between AGN health frontiers and low SDI countries for children and adolescents, revealing avoidable disease burdens. The time trend from 1990 to 2021 demonstrates that as SDI increases, countries generally converge towards the frontier; however, the rates of improvement exhibit significant heterogeneity, highlighting AGN outcome inequalities that extend beyond mere economic development factors. Laos (eff_diff = 16.42) and Timor-Leste (16.03) displayed the most substantial health efficiency losses, with actual DALYs rates over 16 times higher than the theoretical optimal values. This indicates an urgent need for these countries to improve primary healthcare accessibility (38). The Philippines (SDI = 0.65), despite having a relatively higher development level, shows a DALY burden (13.72) significantly exceeding the optimal level (0.03) for its SDI group. This suggests insufficient health system efficiency, implying that middle SDI countries like the Philippines should focus on optimizing resource allocation efficiency rather than simply increasing investments (39). Resource-constrained regions must strengthen their primary healthcare capabilities to sustain declining trends and narrow regional disparities.

The study acknowledges several limitations. First, although the GBD employs complex models, such as data source weighting and Bayesian meta-regression, to adjust for data heterogeneity and incompleteness, inherent variations in diagnostic criteria, ICD coding practices, and surveillance system strength across countries and over time may introduce systematic biases into the incidence and DALY estimates. For instance, in regions with limited diagnostic capacity, case numbers are likely under-ascertained, whereas incidence may be overestimated in areas with centralized specialist referral centers. Crucially, because the GBD provides final, model adjusted estimates rather than raw data streams, it was not feasible to perform country level sensitivity analyses, such as excluding countries with the lowest data quality scores, which is a recognized constraint of using such aggregated data. These issues are particularly critical when interpreting the high incidence rates in low-SDI regions. The observed burden may be influenced by under-ascertainment of mild cases or, conversely, by misclassification of other glomerular diseases as AGN due to the infrequent use of kidney biopsies (26, 40, 41). Nevertheless, the compelling disparity in disease burden across SDI regions, along with the strong alignment between high rates in areas such as Tropical Latin America and the established epidemiology of APSGN (1, 13), suggests the presence of a substantial real disease burden—even though absolute values should be interpreted with caution. Second, our BAPC projections rely on the continuation of historical trends and may not fully account for future disruptions or innovations, such as public health interventions, socioeconomic transitions, or changes in pathogen epidemiology, thereby introducing uncertainty into long-term predictions. Finally, although the SDI offers a valuable macro-level framework, it may not capture all local determinants of the disease. Despite these limitations, the core trends we report, such as the overall global decline in AGN burden and persistent disparities across SDI regions, are robust and provide a systematic foundation for public health planning and future research.

## Conclusion

This study provides a comprehensive assessment of the global burden of AGN among children and adolescents from 1990 to 2021, revealing complex epidemiological characteristics. Despite the overall decline in incidence and DALYs, significant disparities persist due to regional and socioeconomic factors. Regions with a middle SDI have the highest DALYs, while some low-middle-income areas are experiencing a rebound in incidence. China reported the highest number of confirmed AGN cases in 2021, but it also demonstrated the most significant reduction in incidence, contrasting with India and Brazil. Age and gender disparities are also pronounced, with children under 5 years bearing a greater severity of disease. This study highlights the importance of developing targeted public health policies to address the evolving burden of AGN and underscores the urgent need for enhanced early screening and health education in resource-limited settings to reduce health inequities.

## Data Availability

The raw data supporting the conclusions of this article will be made available by the authors, without undue reservation.

## Footnotes

1
https://vizhub.healthdata.org/gbd-results/

2
https://ghdx.healthdata.org/gbd-2021
