# Supplementary material for: Global, regional, and national burden of acute glomerulonephritis in children and adolescents: 1990–2021 analysis and future projections
Source: Front Public Health. 2025 Dec 3;13:1677679. doi: 10.3389/fpubh.2025.1677679 (PMC12708533; doi:10.3389/fpubh.2025.1677679)
Supplement: Supplementary file 1 [file Data_Sheet_1.docx]

**Table S1** Number of death and age-standardized death rates per 100,000 population of acute glomerulonephritis among children and adolescents in 1990 and 2021 and temporal trends.

| Characteristics | 1990 |  | 2021 |  | 1990-2021 |
| --- | --- | --- | --- | --- | --- |
|  | death cases no. (95%UI) | rate/100,000 | death cases no. (95%UI) | rate/100,000 | EAPC no. (95%CI) |
| **Overall** | 2638.61(1884.63,3139.42) | 0.12(0.08,0.14) | 722.35(397.94,968.63) | 0.03(0.02,0.04) | -4.34(-4.46, -4.22) |
| **Sex** |  |  |  |  |  |
| Male | 1477.24(770.47,1803.91) | 0.13(0.07,0.16) | 385.00(135.30,555.54) | 0.03(0.01,0.04) | -4.48(-4.62, -4.34) |
| Female | 1161.37(920.85,1515.40) | 0.11(0.08,0.14) | 337.35(254.23,522.39) | 0.03(0.02,0.04) | -4.16(-4.26, -4.06) |
| **Socio-demographic factor** | |  |  |  |  |
| High SDI | 10.45(8.48,13.41) | 0.00(0.00,0.01) | 5.50(4.43,6.71) | 0.00(0.00,0.00) | -1.67(-2.79, -0.53) |
| High-middle SDI | 379.18(293.45,490.24) | 0.10(0.08,0.13) | 39.36(27.68,56.58) | 0.01(0.01,0.02) | -6.48(-6.57, -6.38) |
| Middle SDI | 1531.10(1110.41,1868.62) | 0.20(0.15,0.24) | 290.33(180.67,398.24) | 0.04(0.02,0.05) | -4.89(-5.04, -4.73) |
| Low-middle SDI | 495.67(262.20,713.83) | 0.08(0.04,0.12) | 164.95(84.69,227.36) | 0.02(0.01,0.03) | -4.15(-4.22, -4.08) |
| Low SDI | 220.79(86.75,346.87) | 0.08(0.03,0.12) | 221.65(93.57,332.33) | 0.04(0.02,0.06) | -2.07(-2.21, -1.93) |
| **Region** |  |  |  |  |  |
| Andean Latin America | 7.34(4.83,9.25) | 0.04(0.03,0.05) | 2.20(1.49,3.20) | 0.01(0.01,0.01) | -4.94(-5.94, -3.94) |
| Australasia | 0.17(0.12,0.23) | 0.00(0.00,0.00) | 0.20(0.12,0.31) | 0.00(0.00,0.00) | 2.26(-0.84, 5.46) |
| Caribbean | 4.10(2.96,5.24) | 0.03(0.02,0.03) | 2.33(1.58,3.30) | 0.02(0.01,0.02) | -1.28(-1.94, -0.61) |
| Central Asia | 25.56(18.91,34.85) | 0.08(0.06,0.11) | 6.69(4.79,9.22) | 0.02(0.01,0.03) | -4.80(-5.33, -4.26) |
| Central Europe | 5.90(4.52,7.51) | 0.02(0.01,0.02) | 2.32(1.88,2.91) | 0.01(0.01,0.01) | -2.08(-3.54, -0.59) |
| Central Latin America | 11.30(9.69,12.89) | 0.01(0.01,0.02) | 33.55(26.43,42.67) | 0.04(0.03,0.05) | 5.16(1.96,8.46) |
| Central Sub-Saharan Africa | 20.53(9.22,31.57) | 0.07(0.03,0.10) | 17.19(7.81,24.81) | 0.02(0.01,0.03) | -2.84(-3.15, -2.53) |
| East Asia | 1312.70(993.90,1692.80) | 0.29(0.22,0.37) | 117.66(75.36,167.08) | 0.03(0.02,0.05) | -6.39(-6.57, -6.20) |
| Eastern Europe | 49.07(42.92,55.46) | 0.07(0.06,0.08) | 2.55(2.16,2.99) | 0.01(0.00,0.01) | -9.11(-9.71, -8.50) |
| Eastern Sub-Saharan Africa | 104.85(35.74,170.12) | 0.09(0.03,0.15) | 89.79(36.06,153.58) | 0.04(0.02,0.07) | -2.55(-2.66, -2.43) |
| High-income Asia Pacific | 0.66(0.51,0.90) | 0.00(0.00,0.00) | 0.17(0.14,0.21) | 0.00(0.00,0.00) | -3.24(-3.54, -2.93) |
| High-income North America | 0.72(0.63,0.81) | 0.00(0.00,0.00) | 1.70(1.39,2.03) | 0.00(0.00,0.00) | 4.61(1.60, 7.71) |
| North Africa and Middle East | 67.40(47.55,85.69) | 0.04(0.03,0.05) | 34.12(18.39,49.59) | 0.01(0.01,0.02) | -1.80(-2.41, -1.18) |
| Oceania | 0.01(0.00,0.04) | 0.00(0.00,0.00) | 0.02(0.01,0.03) | 0.00(0.00,0.00) | -1.86(-2.06, -1.67) |
| South Asia | 72.66(41.77,95.98) | 0.01(0.01,0.02) | 23.55(15.80,33.69) | 0.00(0.00,0.00) | -4.46(-4.73, -4.18) |
| Southeast Asia | 737.46(359.85,1110.37) | 0.34(0.16,0.50) | 238.11(125.17,340.52) | 0.10(0.05,0.15) | -3.47(-3.59, -3.36) |
| Southern Latin America | 0.56(0.39,0.78) | 0.00(0.00,0.00) | 0.55(0.42,0.75) | 0.00(0.00,0.00) | 0.26(-1.53, 2.08) |
| Southern Sub-Saharan Africa | 11.18(7.93,15.82) | 0.04(0.03,0.06) | 10.63(6.95,15.22) | 0.03(0.02,0.05) | -0.10(-0.39, 0.19) |
| Tropical Latin America | 103.26(85.12,122.47) | 0.15(0.12,0.18) | 11.50(8.79,14.20) | 0.02(0.01,0.02) | -6.44(-6.89, -5.98) |
| Western Europe | 1.21(1.04,1.38) | 0.00(0.00,0.00) | 0.90(0.75,1.05) | 0.00(0.00,0.00) | -0.26(-2.45, 1.98) |
| Western Sub-Saharan Africa | 101.97(33.16,169.05) | 0.09(0.03,0.16) | 126.61(50.97,186.59) | 0.05(0.02,0.07) | -1.74(-1.95, -1.52) |

**Table S2** The incidence of AGN in children and adolescents in 204 countries or territories in 2021 (Related to Figure 1).

|  | **Incidence Number (95%UI)** | **ASIR / 100,000(95%UI)** | **EAPC (95%CI)** |
| --- | --- | --- | --- |
| Afghanistan | 1195.23(831.35,1612.69) | 6.72(4.68,9.07) | 1.06(0.96,1.16) |
| Albania | 62.29(38.93,87.72) | 10.09(6.31,14.21) | -0.85(-0.88,-0.81) |
| Algeria | 916.25(574.59,1246.65) | 5.57(3.49,7.58) | 0.37(0.28,0.46) |
| American Samoa | 2.24(1.40,3.17) | 11.69(7.31,16.59) | 0.14(0.09,0.20) |
| Andorra | 0.40(0.24,0.60) | 2.77(1.65,4.15) | 0.01(-0.01,0.03) |
| Angola | 992.09(636.73,1376.73) | 5.31(3.41,7.37) | -0.41(-0.45,-0.38) |
| Antigua and Barbuda | 1.47(0.93,2.08) | 6.29(3.98,8.89) | 0.13(0.06,0.21) |
| Argentina | 278.36(170.24,406.25) | 2.03(1.24,2.96) | 0.02(-0.04,0.07) |
| Armenia | 87.68(55.02,123.64) | 11.49(7.21,16.20) | 0.60(0.44,0.75) |
| Australia | 167.27(101.69,246.61) | 2.68(1.63,3.95) | 0.01(-0.02,0.03) |
| Austria | 120.95(83.64,165.69) | 6.89(4.77,9.44) | -0.46(-0.68,-0.23) |
| Azerbaijan | 298.80(182.25,428.92) | 9.81(5.98,14.08) | 0.35(0.20,0.50) |
| Bahamas | 8.10(5.03,11.43) | 7.03(4.36,9.92) | 0.42(0.40,0.43) |
| Bahrain | 22.31(14.11,31.20) | 5.53(3.50,7.73) | 0.46(0.41,0.51) |
| Bangladesh | 1616.21(992.03,2323.08) | 2.65(1.63,3.81) | 0.16(0.13,0.18) |
| Barbados | 4.19(2.63,5.90) | 6.31(3.95,8.89) | 0.15(0.09,0.22) |
| Belarus | 164.64(100.53,235.15) | 8.13(4.96,11.61) | -0.03(-0.18,0.12) |
| Belgium | 84.11(52.12,124.34) | 3.31(2.05,4.89) | 0.21(-0.18,0.60) |
| Belize | 12.15(7.64,16.87) | 7.19(4.52,9.98) | 0.25(0.20,0.31) |
| Benin | 227.03(143.07,319.17) | 3.02(1.90,4.25) | 0.15(0.10,0.21) |
| Bermuda | 0.73(0.45,1.05) | 6.38(3.94,9.10) | 0.18(0.12,0.24) |
| Bhutan | 6.78(4.17,9.61) | 2.66(1.63,3.77) | 0.12(0.07,0.17) |
| Bolivia (Plurinational State of) | 232.64(144.50,333.53) | 5.12(3.18,7.34) | -0.52(-0.56,-0.48) |
| Bosnia and Herzegovina | 65.22(40.95,92.31) | 9.81(6.16,13.88) | -0.09(-0.18,-0.00) |
| Botswana | 37.38(23.25,52.61) | 4.08(2.54,5.74) | 0.03(0.01,0.06) |
| Brazil | 20630.85(16065.72,25893.64) | 32.28(25.14,40.51) | 4.11(3.61,4.62) |
| Brunei Darussalam | 14.20(9.39,20.35) | 11.00(7.27,15.76) | -0.07(-0.18,0.03) |
| Bulgaria | 127.74(78.40,183.67) | 9.87(6.06,14.19) | -0.28(-0.34,-0.23) |
| Burkina Faso | 384.02(240.06,542.24) | 3.00(1.87,4.23) | 0.13(0.07,0.18) |
| Burundi | 374.97(242.91,517.96) | 5.15(3.34,7.12) | 0.13(0.07,0.18) |
| Cabo Verde | 6.32(3.86,9.07) | 3.28(2.00,4.71) | 0.28(0.20,0.36) |
| Cambodia | 1078.82(758.95,1476.91) | 16.27(11.45,22.27) | -1.04(-1.14,-0.95) |
| Cameroon | 519.64(325.49,741.78) | 3.07(1.93,4.39) | 0.13(0.08,0.19) |
| Canada | 138.62(82.66,204.49) | 1.68(1.00,2.49) | -0.03(-0.09,0.02) |
| Central African Republic | 158.91(104.93,219.73) | 5.49(3.63,7.59) | 0.13(0.11,0.16) |
| Chad | 322.48(200.87,454.49) | 2.95(1.83,4.15) | 0.09(0.03,0.14) |
| Chile | 166.23(104.89,248.73) | 3.40(2.14,5.08) | 1.60(1.27,1.93) |
| China | 29607.98(21973.98,39040.17) | 8.86(6.57,11.68) | -2.56(-2.81,-2.31) |
| Colombia | 1205.66(766.23,1666.29) | 8.28(5.26,11.44) | -0.24(-0.29,-0.19) |
| Comoros | 16.09(10.14,22.21) | 5.13(3.23,7.07) | 0.22(0.19,0.25) |
| Congo | 130.24(81.99,181.85) | 5.24(3.30,7.31) | 0.09(0.06,0.11) |
| Cook Islands | 0.61(0.39,0.86) | 11.76(7.45,16.68) | 0.09(0.04,0.14) |
| Costa Rica | 114.80(72.16,162.45) | 8.39(5.27,11.87) | -0.22(-0.26,-0.19) |
| Croatia | 95.43(63.69,130.15) | 11.84(7.90,16.15) | -0.54(-0.59,-0.48) |
| Cuba | 154.13(97.09,217.62) | 6.42(4.04,9.06) | 0.03(-0.04,0.10) |
| Cyprus | 3.53(2.14,5.32) | 1.24(0.75,1.87) | 0.13(-0.06,0.33) |
| Czechia | 225.35(143.52,315.41) | 10.15(6.46,14.20) | -0.31(-0.37,-0.25) |
| Côte d'Ivoire | 434.52(273.71,610.83) | 3.02(1.90,4.25) | 0.06(0.00,0.12) |
| Democratic People's Republic of Korea | 1932.37(1288.62,2638.75) | 29.27(19.52,39.97) | 0.56(0.50,0.61) |
| Democratic Republic of the Congo | 2587.54(1670.37,3554.98) | 5.40(3.49,7.42) | 0.05(0.03,0.08) |
| Denmark | 37.55(23.22,55.08) | 2.90(1.79,4.26) | -0.10(-0.57,0.36) |
| Djibouti | 25.95(16.52,36.50) | 4.91(3.13,6.91) | -0.04(-0.08,0.00) |
| Dominica | 1.25(0.78,1.79) | 6.46(4.01,9.24) | 0.25(0.17,0.32) |
| Dominican Republic | 236.97(149.63,330.42) | 6.09(3.85,8.49) | 0.08(-0.01,0.17) |
| Ecuador | 562.29(406.31,760.77) | 8.50(6.14,11.50) | 1.96(1.68,2.24) |
| Egypt | 2568.80(1670.28,3565.55) | 5.52(3.59,7.66) | 0.39(0.33,0.44) |
| El Salvador | 198.40(127.10,278.08) | 8.33(5.34,11.67) | -0.21(-0.29,-0.13) |
| Equatorial Guinea | 39.79(24.64,55.60) | 5.17(3.20,7.22) | 0.03(0.00,0.05) |
| Eritrea | 160.71(102.60,220.82) | 5.00(3.19,6.87) | 0.11(0.09,0.13) |
| Estonia | 22.67(13.82,32.33) | 8.09(4.93,11.54) | 0.05(-0.07,0.18) |
| Eswatini | 21.69(13.76,30.21) | 4.05(2.57,5.65) | 0.04(-0.00,0.09) |
| Ethiopia | 2724.75(2113.45,3449.67) | 4.76(3.69,6.03) | -0.63(-0.69,-0.57) |
| Fiji | 39.73(24.92,56.13) | 11.33(7.11,16.01) | -0.07(-0.14,-0.00) |
| Finland | 55.99(35.29,81.55) | 4.87(3.07,7.10) | -0.07(-0.15,0.02) |
| France | 426.86(261.37,634.17) | 2.71(1.66,4.03) | -0.05(-0.08,-0.02) |
| Gabon | 43.48(27.32,59.99) | 5.26(3.30,7.25) | 0.19(0.16,0.22) |
| Gambia | 39.13(24.54,55.06) | 3.09(1.94,4.35) | 0.15(0.10,0.19) |
| Georgia | 88.85(60.88,123.76) | 9.56(6.55,13.32) | 0.75(0.61,0.89) |
| Germany | 209.18(125.28,310.28) | 1.31(0.79,1.95) | 0.65(0.08,1.22) |
| Ghana | 500.39(308.85,703.94) | 3.07(1.89,4.31) | 0.16(0.09,0.22) |
| Greece | 20.43(12.42,30.99) | 1.07(0.65,1.63) | -0.16(-0.18,-0.14) |
| Greenland | 0.25(0.15,0.35) | 1.61(0.99,2.31) | 0.01(-0.07,0.10) |
| Grenada | 1.87(1.18,2.64) | 6.21(3.92,8.76) | 0.13(0.05,0.21) |
| Guam | 5.61(3.54,7.86) | 11.57(7.30,16.21) | 0.09(0.02,0.15) |
| Guatemala | 538.36(346.82,756.44) | 8.14(5.24,11.44) | -0.26(-0.35,-0.18) |
| Guinea | 223.78(139.05,311.74) | 3.00(1.86,4.18) | 0.15(0.08,0.22) |
| Guinea-Bissau | 33.62(21.11,47.66) | 3.01(1.89,4.26) | 0.02(-0.04,0.08) |
| Guyana | 17.69(11.37,24.57) | 6.30(4.05,8.75) | 0.27(0.18,0.37) |
| Haiti | 348.80(231.29,482.38) | 6.22(4.12,8.60) | 0.16(0.10,0.21) |
| Honduras | 346.37(220.75,481.08) | 7.96(5.07,11.06) | -0.26(-0.34,-0.17) |
| Hungary | 186.95(118.29,262.44) | 9.97(6.31,13.99) | -0.21(-0.25,-0.17) |
| Iceland | 0.96(0.57,1.44) | 1.07(0.64,1.62) | 0.01(-0.05,0.07) |
| India | 13514.28(10327.76,17251.58) | 2.70(2.06,3.45) | 0.20(0.14,0.26) |
| Indonesia | 8962.55(6662.15,11736.66) | 9.93(7.38,13.01) | -1.52(-1.63,-1.41) |
| Iran (Islamic Republic of) | 2959.52(2363.05,3718.80) | 11.45(9.14,14.38) | 2.21(1.89,2.52) |
| Iraq | 949.45(600.57,1340.14) | 5.38(3.41,7.60) | 0.33(0.29,0.37) |
| Ireland | 35.84(22.32,53.79) | 2.71(1.69,4.07) | -0.12(-0.14,-0.10) |
| Israel | 89.42(56.30,131.00) | 2.65(1.67,3.88) | -0.13(-0.16,-0.11) |
| Italy | 1265.95(984.09,1593.06) | 12.08(9.39,15.20) | 1.58(1.32,1.83) |
| Jamaica | 51.33(31.64,72.82) | 6.30(3.88,8.93) | 0.18(0.09,0.26) |
| Japan | 3664.57(2771.64,4753.96) | 17.26(13.05,22.39) | 2.25(1.76,2.74) |
| Jordan | 275.69(173.19,393.09) | 5.60(3.52,7.98) | 0.43(0.41,0.45) |
| Kazakhstan | 602.65(368.53,853.37) | 8.96(5.48,12.69) | 0.10(-0.08,0.28) |
| Kenya | 1215.55(929.64,1537.90) | 4.94(3.78,6.25) | 0.01(-0.04,0.06) |
| Kiribati | 5.89(3.72,8.21) | 11.02(6.97,15.37) | 0.00(-0.09,0.10) |
| Kuwait | 61.37(38.37,85.68) | 5.60(3.50,7.82) | 0.42(0.37,0.47) |
| Kyrgyzstan | 278.31(178.70,395.53) | 9.86(6.33,14.01) | -0.19(-0.28,-0.10) |
| Lao People's Democratic Republic | 530.90(375.38,706.24) | 17.75(12.55,23.61) | -0.62(-0.69,-0.55) |
| Latvia | 31.21(19.29,44.84) | 8.06(4.98,11.58) | 0.00(-0.17,0.17) |
| Lebanon | 94.26(59.63,130.25) | 5.66(3.58,7.82) | 0.45(0.40,0.51) |
| Lesotho | 34.32(21.48,48.80) | 4.09(2.56,5.82) | 0.11(0.08,0.13) |
| Liberia | 85.80(54.35,120.38) | 3.08(1.95,4.31) | 0.14(0.09,0.18) |
| Libya | 119.38(74.37,168.20) | 5.72(3.56,8.06) | 0.49(0.45,0.53) |
| Lithuania | 43.14(25.76,61.26) | 8.05(4.81,11.43) | 0.27(0.14,0.40) |
| Luxembourg | 2.18(1.34,3.25) | 1.62(0.99,2.41) | 0.62(0.09,1.15) |
| Madagascar | 825.85(530.85,1131.53) | 5.53(3.55,7.57) | 0.14(0.05,0.23) |
| Malawi | 550.51(346.04,768.45) | 5.20(3.27,7.26) | -0.02(-0.10,0.07) |
| Malaysia | 1442.24(920.54,1998.43) | 14.06(8.98,19.49) | -0.01(-0.06,0.04) |
| Maldives | 18.57(11.90,26.34) | 14.25(9.14,20.21) | -0.01(-0.06,0.05) |
| Mali | 427.12(265.20,596.48) | 3.00(1.86,4.19) | 0.11(0.07,0.16) |
| Malta | 1.62(0.99,2.41) | 1.93(1.17,2.87) | -0.18(-0.21,-0.15) |
| Marshall Islands | 2.57(1.65,3.62) | 11.14(7.14,15.69) | -0.04(-0.11,0.02) |
| Mauritania | 72.67(44.94,102.54) | 3.12(1.93,4.40) | 0.18(0.13,0.23) |
| Mauritius | 42.03(26.76,58.46) | 14.16(9.01,19.69) | -0.00(-0.06,0.06) |
| Mexico | 2202.30(1440.90,3114.20) | 5.10(3.34,7.22) | -1.20(-1.45,-0.95) |
| Micronesia (Federated States of) | 4.70(2.95,6.65) | 11.33(7.13,16.04) | 0.03(-0.05,0.10) |
| Monaco | 0.18(0.11,0.27) | 2.68(1.61,3.97) | -0.07(-0.13,-0.01) |
| Mongolia | 112.09(68.01,159.70) | 8.54(5.18,12.17) | 0.13(-0.08,0.34) |
| Montenegro | 14.79(9.29,20.87) | 9.84(6.18,13.88) | -0.11(-0.16,-0.05) |
| Morocco | 733.33(474.31,1007.64) | 5.68(3.67,7.80) | 0.44(0.39,0.50) |
| Mozambique | 970.39(630.63,1330.47) | 5.47(3.55,7.49) | -0.32(-0.40,-0.23) |
| Myanmar | 3614.72(2535.42,4837.20) | 17.41(12.21,23.29) | -0.79(-0.87,-0.70) |
| Namibia | 42.91(26.61,60.57) | 4.01(2.48,5.65) | 0.04(0.00,0.08) |
| Nauru | 0.57(0.37,0.80) | 11.19(7.24,15.52) | 0.03(-0.02,0.07) |
| Nepal | 325.98(205.53,460.75) | 2.62(1.65,3.70) | 0.22(0.17,0.26) |
| Netherlands | 98.90(61.32,145.72) | 2.68(1.66,3.95) | -0.03(-0.06,0.01) |
| New Zealand | 89.74(70.80,112.97) | 6.88(5.43,8.67) | -0.45(-0.83,-0.07) |
| Nicaragua | 209.83(133.58,295.17) | 8.07(5.14,11.36) | -0.29(-0.35,-0.24) |
| Niger | 457.73(289.73,645.73) | 2.94(1.86,4.14) | 0.12(0.06,0.17) |
| Nigeria | 4068.81(3185.34,5172.08) | 3.18(2.49,4.05) | 0.10(0.05,0.16) |
| Niue | 0.06(0.04,0.09) | 11.70(7.37,16.39) | 0.04(-0.01,0.10) |
| North Macedonia | 43.43(27.02,60.17) | 9.73(6.05,13.47) | -0.10(-0.17,-0.02) |
| Northern Mariana Islands | 1.77(1.12,2.49) | 11.78(7.48,16.63) | 0.21(0.15,0.27) |
| Norway | 55.08(41.76,71.03) | 4.44(3.36,5.72) | 0.17(0.03,0.32) |
| Oman | 84.00(54.08,115.96) | 5.63(3.63,7.77) | 0.41(0.33,0.50) |
| Pakistan | 2836.75(2193.73,3627.07) | 2.60(2.01,3.32) | 0.11(0.04,0.18) |
| Palau | 0.52(0.32,0.73) | 11.66(7.28,16.43) | 0.02(-0.03,0.08) |
| Palestine | 132.66(84.63,183.79) | 5.51(3.51,7.63) | 0.45(0.40,0.50) |
| Panama | 126.07(80.43,177.02) | 8.36(5.34,11.74) | -0.15(-0.21,-0.09) |
| Papua New Guinea | 529.72(344.72,721.22) | 10.81(7.04,14.72) | -0.07(-0.17,0.02) |
| Paraguay | 222.78(140.99,314.54) | 8.34(5.28,11.78) | -0.30(-0.34,-0.27) |
| Peru | 662.85(407.36,948.94) | 5.30(3.26,7.59) | -0.25(-0.29,-0.21) |
| Philippines | 6755.35(5335.33,8537.71) | 15.09(11.92,19.07) | -1.75(-2.09,-1.40) |
| Poland | 789.52(606.81,1007.06) | 10.26(7.88,13.08) | -0.21(-0.27,-0.15) |
| Portugal | 38.08(23.30,56.41) | 1.99(1.22,2.94) | 0.00(-0.04,0.04) |
| Puerto Rico | 42.00(25.58,60.55) | 6.43(3.91,9.27) | 0.22(0.17,0.27) |
| Qatar | 32.32(20.87,45.20) | 5.41(3.49,7.57) | 0.40(0.35,0.45) |
| Republic of Korea | 976.50(620.06,1421.50) | 11.65(7.40,16.96) | 0.02(-0.05,0.08) |
| Republic of Moldova | 56.77(34.72,81.68) | 8.15(4.99,11.73) | 0.24(0.08,0.41) |
| Romania | 425.42(267.24,601.37) | 10.53(6.61,14.88) | -0.08(-0.22,0.07) |
| Russian Federation | 3029.19(2295.23,3867.26) | 8.96(6.79,11.45) | -1.08(-1.24,-0.93) |
| Rwanda | 329.74(210.25,461.96) | 5.12(3.27,7.18) | -0.16(-0.21,-0.11) |
| Saint Kitts and Nevis | 0.90(0.57,1.25) | 6.39(4.03,8.88) | 0.23(0.16,0.30) |
| Saint Lucia | 2.62(1.64,3.67) | 6.25(3.92,8.75) | 0.14(0.06,0.22) |
| Saint Vincent and the Grenadines | 2.29(1.41,3.19) | 6.79(4.19,9.48) | 0.25(0.21,0.28) |
| Samoa | 11.32(7.22,15.77) | 11.21(7.15,15.62) | -0.06(-0.12,0.00) |
| San Marino | 0.18(0.11,0.27) | 2.91(1.80,4.30) | 0.04(0.01,0.08) |
| Sao Tome and Principe | 3.24(1.99,4.55) | 3.18(1.95,4.47) | 0.18(0.13,0.22) |
| Saudi Arabia | 561.79(349.00,783.22) | 5.54(3.44,7.73) | 0.47(0.41,0.54) |
| Senegal | 249.85(154.00,353.75) | 3.08(1.90,4.36) | 0.16(0.12,0.21) |
| Serbia | 186.77(115.14,268.20) | 9.97(6.14,14.31) | -0.18(-0.33,-0.04) |
| Seychelles | 4.37(2.85,6.07) | 14.30(9.33,19.90) | -0.01(-0.04,0.03) |
| Sierra Leone | 141.93(91.00,199.41) | 3.12(2.00,4.38) | 0.19(0.14,0.25) |
| Singapore | 115.81(75.19,165.70) | 11.10(7.21,15.88) | -0.04(-0.15,0.08) |
| Slovakia | 102.28(65.04,145.32) | 9.12(5.80,12.96) | 0.15(-0.11,0.41) |
| Slovenia | 31.42(19.30,44.32) | 7.76(4.77,10.95) | -0.14(-0.23,-0.04) |
| Solomon Islands | 36.35(23.40,49.92) | 10.99(7.08,15.09) | -0.04(-0.09,0.01) |
| Somalia | 659.54(440.05,895.26) | 5.16(3.44,7.00) | -0.13(-0.20,-0.06) |
| South Africa | 851.05(653.40,1077.18) | 4.27(3.28,5.41) | -0.25(-0.34,-0.17) |
| South Sudan | 280.87(182.97,390.72) | 5.12(3.33,7.12) | 0.06(-0.00,0.13) |
| Spain | 234.70(149.02,356.45) | 2.66(1.69,4.05) | 0.82(0.63,1.02) |
| Sri Lanka | 988.60(620.34,1392.83) | 14.33(8.99,20.19) | 0.02(-0.02,0.06) |
| Sudan | 1242.63(801.11,1703.14) | 5.81(3.75,7.97) | 0.71(0.65,0.76) |
| Suriname | 12.58(8.05,17.63) | 6.63(4.24,9.29) | 0.23(0.20,0.25) |
| Sweden | 193.43(144.38,252.44) | 8.01(5.98,10.45) | 0.77(-0.03,1.59) |
| Switzerland | 53.73(33.70,79.65) | 3.05(1.91,4.52) | -0.25(-0.54,0.04) |
| Syrian Arab Republic | 307.22(191.69,435.34) | 5.65(3.52,8.00) | 0.51(0.47,0.55) |
| Taiwan (Province of China) | 1043.17(587.62,1446.02) | 25.79(14.53,35.74) | -1.85(-2.39,-1.31) |
| Tajikistan | 385.76(237.72,553.14) | 8.66(5.34,12.42) | 0.45(0.30,0.60) |
| Thailand | 1922.46(1224.51,2748.03) | 14.09(8.97,20.13) | 0.01(-0.05,0.07) |
| Timor-Leste | 101.25(69.81,137.63) | 14.77(10.18,20.08) | -0.41(-0.45,-0.37) |
| Togo | 127.63(80.18,180.88) | 3.06(1.92,4.33) | 0.14(0.09,0.19) |
| Tokelau | 0.06(0.04,0.08) | 11.63(7.50,16.50) | 0.05(-0.01,0.11) |
| Tonga | 5.55(3.51,7.69) | 11.19(7.08,15.51) | -0.02(-0.09,0.04) |
| Trinidad and Tobago | 22.71(14.11,32.07) | 6.24(3.88,8.81) | 0.08(0.02,0.14) |
| Tunisia | 200.72(127.15,278.95) | 5.60(3.55,7.78) | 0.38(0.31,0.45) |
| Turkmenistan | 190.51(118.45,265.94) | 9.72(6.04,13.56) | 0.22(0.15,0.29) |
| Tuvalu | 0.55(0.35,0.78) | 11.21(7.09,15.81) | 0.13(0.05,0.21) |
| Turkey | 1364.69(857.24,1934.59) | 5.52(3.47,7.83) | 0.42(0.37,0.47) |
| Uganda | 1244.36(797.37,1740.48) | 4.99(3.20,6.98) | -0.02(-0.06,0.02) |
| Ukraine | 775.80(580.34,1007.82) | 9.19(6.87,11.93) | 0.19(0.06,0.33) |
| United Arab Emirates | 94.40(59.43,131.03) | 5.57(3.51,7.73) | 0.43(0.36,0.51) |
| United Kingdom | 510.05(319.50,757.63) | 3.26(2.04,4.84) | -0.83(-1.01,-0.64) |
| United Republic of Tanzania | 1613.04(1052.49,2241.72) | 5.23(3.41,7.26) | 0.02(-0.03,0.07) |
| United States Virgin Islands | 1.17(0.73,1.64) | 6.44(3.98,8.98) | 0.11(0.06,0.16) |
| United States of America | 935.96(742.86,1154.39) | 1.15(0.91,1.42) | -1.05(-1.14,-0.95) |
| Uruguay | 18.44(11.31,27.00) | 2.04(1.25,2.99) | 0.07(0.02,0.12) |
| Uzbekistan | 1305.96(840.83,1810.55) | 10.36(6.67,14.36) | -0.22(-0.43,-0.01) |
| Vanuatu | 16.03(10.41,22.26) | 10.87(7.06,15.10) | -0.08(-0.16,-0.01) |
| Venezuela (Bolivarian Republic of) | 713.85(453.01,989.35) | 8.16(5.18,11.30) | -0.19(-0.24,-0.14) |
| Viet Nam | 4551.10(3016.40,6368.90) | 14.39(9.54,20.14) | -0.07(-0.11,-0.03) |
| Yemen | 1013.23(662.84,1384.80) | 5.80(3.79,7.93) | 0.66(0.62,0.70) |
| Zambia | 531.36(340.51,737.76) | 5.08(3.26,7.06) | -0.02(-0.06,0.03) |
| Zimbabwe | 313.19(197.57,436.30) | 3.92(2.47,5.46) | -0.05(-0.09,-0.02) |

Notes: ASIR/100,000: age-standardized incidence rate (per 100,000 population);

**Table S3.** The DALYs of AGN in children and adolescents in 204 countries or territories in 2021.

|  | **DALYs Number (95%UI)** | **AS-DALY rate/100,000(95%UI)** | **EAPC (95%CI)** |
| --- | --- | --- | --- |
| Afghanistan | 889.02(359.92,1478.99) | 5.00(2.02,8.32) | -0.84(-1.48,-0.20) |
| Albania | 15.65(8.00,32.65) | 2.54(1.30,5.29) | -5.53(-5.90,-5.16) |
| Algeria | 94.14(41.50,161.66) | 0.57(0.25,0.98) | -1.62(-2.13,-1.12) |
| American Samoa | 0.01(0.00,0.01) | 0.04(0.02,0.07) | -0.26(-0.31,-0.22) |
| Andorra | 0.00(0.00,0.01) | 0.02(0.01,0.04) | -2.53(-2.92,-2.14) |
| Angola | 424.52(157.66,700.06) | 2.27(0.84,3.75) | -3.90(-4.27,-3.52) |
| Antigua and Barbuda | 0.01(0.00,0.01) | 0.03(0.02,0.04) | -0.20(-0.47,0.07) |
| Argentina | 29.38(20.88,41.51) | 0.21(0.15,0.30) | 0.68(-0.99,2.37) |
| Armenia | 1.45(0.90,2.26) | 0.19(0.12,0.30) | -4.57(-4.99,-4.16) |
| Australia | 16.71(10.48,25.38) | 0.27(0.17,0.41) | 2.06(-0.77,4.97) |
| Austria | 1.70(1.34,2.17) | 0.10(0.08,0.12) | 0.10(-0.99,1.20) |
| Azerbaijan | 31.62(17.13,56.28) | 1.04(0.56,1.85) | -5.48(-5.78,-5.18) |
| Bahamas | 2.52(1.56,4.10) | 2.19(1.35,3.56) | 0.57(-0.39,1.54) |
| Bahrain | 0.91(0.42,1.88) | 0.23(0.10,0.46) | -2.57(-2.92,-2.20) |
| Bangladesh | 210.15(136.10,296.10) | 0.34(0.22,0.49) | -3.46(-3.58,-3.33) |
| Barbados | 0.01(0.01,0.03) | 0.02(0.01,0.04) | 0.22(0.17,0.27) |
| Belarus | 5.06(3.15,7.98) | 0.25(0.16,0.39) | -5.35(-5.72,-4.99) |
| Belgium | 1.63(1.27,2.10) | 0.06(0.05,0.08) | -0.04(-1.16,1.10) |
| Belize | 3.63(2.41,5.16) | 2.15(1.43,3.05) | -2.40(-3.49,-1.31) |
| Benin | 310.40(121.72,517.32) | 4.13(1.62,6.88) | -2.45(-2.63,-2.28) |
| Bermuda | 0.00(0.00,0.01) | 0.03(0.02,0.05) | 0.51(0.30,0.72) |
| Bhutan | 0.85(0.45,1.81) | 0.33(0.18,0.71) | -4.34(-4.59,-4.08) |
| Bolivia (Plurinational State of) | 47.56(27.91,70.89) | 1.05(0.61,1.56) | -4.84(-5.12,-4.56) |
| Bosnia and Herzegovina | 0.71(0.44,1.09) | 0.11(0.07,0.16) | -0.73(-1.25,-0.20) |
| Botswana | 24.54(14.94,38.56) | 2.68(1.63,4.20) | -0.95(-1.26,-0.63) |
| Brazil | 987.11(772.49,1202.99) | 1.54(1.21,1.88) | -6.33(-6.80,-5.87) |
| Brunei Darussalam | 0.19(0.12,0.28) | 0.15(0.10,0.22) | -2.63(-3.02,-2.25) |
| Bulgaria | 8.33(5.45,12.31) | 0.64(0.42,0.95) | -2.18(-3.42,-0.92) |
| Burkina Faso | 682.24(207.34,1226.06) | 5.33(1.62,9.57) | -1.46(-1.64,-1.28) |
| Burundi | 264.32(99.08,497.97) | 3.63(1.36,6.85) | -2.28(-2.59,-1.96) |
| Cabo Verde | 1.64(0.57,3.97) | 0.85(0.30,2.06) | -4.19(-4.59,-3.79) |
| Cambodia | 824.18(280.38,1547.15) | 12.43(4.23,23.33) | -5.24(-5.47,-5.02) |
| Cameroon | 419.91(183.51,634.58) | 2.48(1.09,3.75) | -1.64(-1.98,-1.29) |
| Canada | 2.61(1.96,3.38) | 0.03(0.02,0.04) | 2.85(1.17,4.57) |
| Central African Republic | 137.21(61.04,224.88) | 4.74(2.11,7.77) | -1.46(-1.59,-1.33) |
| Chad | 806.23(265.69,1318.83) | 7.36(2.43,12.04) | -0.66(-0.84,-0.49) |
| Chile | 11.09(7.81,16.22) | 0.23(0.16,0.33) | -0.10(-1.99,1.82) |
| China | 9069.28(5865.97,13070.27) | 2.71(1.75,3.91) | -6.55(-6.72,-6.37) |
| Colombia | 322.60(229.61,429.90) | 2.22(1.58,2.95) | 4.47(1.55,7.48) |
| Comoros | 8.12(4.36,12.74) | 2.59(1.39,4.06) | -2.52(-2.90,-2.13) |
| Congo | 34.35(15.00,57.45) | 1.38(0.60,2.31) | -3.10(-3.43,-2.76) |
| Cook Islands | 0.00(0.00,0.00) | 0.04(0.02,0.07) | -0.24(-0.29,-0.20) |
| Costa Rica | 8.88(6.78,12.15) | 0.65(0.49,0.89) | 5.45(2.92,8.05) |
| Croatia | 1.23(0.90,1.63) | 0.15(0.11,0.20) | -2.46(-3.51,-1.39) |
| Cuba | 10.74(7.78,14.69) | 0.45(0.32,0.61) | -2.09(-3.52,-0.65) |
| Cyprus | 0.16(0.10,0.25) | 0.06(0.03,0.09) | -1.68(-2.14,-1.21) |
| Czechia | 11.11(7.17,16.63) | 0.50(0.32,0.75) | -2.01(-3.70,-0.29) |
| Côte d'Ivoire | 436.34(156.20,786.44) | 3.03(1.09,5.47) | -1.28(-1.57,-0.99) |
| Democratic People's Republic of Korea | 295.02(121.97,513.55) | 4.47(1.85,7.78) | -2.24(-2.52,-1.96) |
| Democratic Republic of the Congo | 802.61(364.57,1320.37) | 1.68(0.76,2.76) | -2.70(-3.03,-2.37) |
| Denmark | 2.08(1.49,2.87) | 0.16(0.11,0.22) | -0.38(-1.87,1.14) |
| Djibouti | 11.41(4.66,23.47) | 2.16(0.88,4.44) | -2.54(-2.89,-2.19) |
| Dominica | 0.04(0.02,0.07) | 0.22(0.12,0.37) | 2.29(1.66,2.92) |
| Dominican Republic | 25.78(14.29,43.18) | 0.66(0.37,1.11) | -3.80(-4.49,-3.11) |
| Ecuador | 86.34(61.12,123.76) | 1.31(0.92,1.87) | -4.35(-6.51,-2.13) |
| Egypt | 692.12(441.22,1079.54) | 1.49(0.95,2.32) | -3.17(-3.67,-2.67) |
| El Salvador | 2.78(1.52,4.87) | 0.12(0.06,0.20) | -1.76(-2.74,-0.76) |
| Equatorial Guinea | 6.33(2.41,11.85) | 0.82(0.31,1.54) | -7.42(-7.78,-7.07) |
| Eritrea | 133.48(54.49,226.04) | 4.15(1.69,7.03) | -1.92(-2.08,-1.76) |
| Estonia | 0.36(0.26,0.50) | 0.13(0.09,0.18) | -9.98(-11.06,-8.89) |
| Eswatini | 19.86(12.12,31.51) | 3.71(2.27,5.89) | -1.07(-1.25,-0.90) |
| Ethiopia | 2026.11(736.87,4504.77) | 3.54(1.29,7.88) | -3.98(-4.08,-3.88) |
| Fiji | 0.13(0.07,0.24) | 0.04(0.02,0.07) | -0.16(-0.25,-0.08) |
| Finland | 1.76(1.31,2.29) | 0.15(0.11,0.20) | 0.50(-0.89,1.91) |
| France | 5.36(4.11,7.07) | 0.03(0.03,0.04) | -0.31(-1.37,0.77) |
| Gabon | 8.15(2.81,13.70) | 0.99(0.34,1.66) | -2.26(-2.51,-2.02) |
| Gambia | 34.51(14.61,64.77) | 2.72(1.15,5.11) | -2.51(-2.79,-2.23) |
| Georgia | 4.21(2.53,6.46) | 0.45(0.27,0.69) | -11.18(-12.30,-10.05) |
| Germany | 9.49(7.07,12.28) | 0.06(0.04,0.08) | 0.70(-0.81,2.23) |
| Ghana | 262.72(114.85,387.20) | 1.61(0.70,2.37) | -2.48(-2.67,-2.30) |
| Greece | 0.82(0.61,1.08) | 0.04(0.03,0.06) | 1.98(0.42,3.56) |
| Greenland | 0.00(0.00,0.00) | 0.01(0.00,0.01) | -0.67(-0.88,-0.46) |
| Grenada | 0.36(0.21,0.56) | 1.19(0.70,1.85) | 1.35(0.11,2.61) |
| Guam | 0.02(0.01,0.03) | 0.04(0.02,0.07) | -0.19(-0.26,-0.13) |
| Guatemala | 139.86(98.43,195.58) | 2.11(1.49,2.96) | 5.23(2.35,8.19) |
| Guinea | 363.79(136.14,584.20) | 4.87(1.82,7.83) | -2.66(-2.88,-2.44) |
| Guinea-Bissau | 47.52(17.88,81.19) | 4.25(1.60,7.26) | -2.75(-3.14,-2.36) |
| Guyana | 5.21(3.42,8.01) | 1.86(1.22,2.85) | 5.65(3.78,7.55) |
| Haiti | 106.88(56.00,188.88) | 1.91(1.00,3.37) | -1.48(-1.90,-1.07) |
| Honduras | 24.00(11.71,42.18) | 0.55(0.27,0.97) | -3.92(-4.43,-3.41) |
| Hungary | 12.42(7.91,19.01) | 0.66(0.42,1.01) | -1.48(-3.24,0.31) |
| Iceland | 0.05(0.03,0.07) | 0.05(0.04,0.08) | 0.38(-1.20,1.98) |
| India | 1081.60(686.34,1575.69) | 0.22(0.14,0.31) | -5.34(-5.77,-4.90) |
| Indonesia | 7492.62(3565.92,12382.98) | 8.31(3.95,13.73) | -3.21(-3.41,-3.01) |
| Iran (Islamic Republic of) | 198.91(94.71,339.45) | 0.77(0.37,1.31) | -2.57(-4.15,-0.97) |
| Iraq | 59.32(36.06,92.47) | 0.34(0.20,0.52) | -4.54(-4.96,-4.11) |
| Ireland | 0.61(0.44,0.81) | 0.05(0.03,0.06) | -1.89(-2.61,-1.18) |
| Israel | 2.08(1.55,2.75) | 0.06(0.05,0.08) | 0.14(-1.15,1.44) |
| Italy | 5.98(4.15,8.67) | 0.06(0.04,0.08) | -3.98(-4.82,-3.12) |
| Jamaica | 3.68(2.42,5.49) | 0.45(0.30,0.67) | 0.51(-0.19,1.21) |
| Japan | 23.61(17.73,32.49) | 0.11(0.08,0.15) | -0.29(-0.56,-0.02) |
| Jordan | 2.83(1.73,4.39) | 0.06(0.04,0.09) | -5.98(-6.51,-5.45) |
| Kazakhstan | 47.09(28.13,78.96) | 0.70(0.42,1.17) | -5.66(-6.42,-4.90) |
| Kenya | 350.12(163.34,577.01) | 1.42(0.66,2.35) | -1.24(-1.71,-0.78) |
| Kiribati | 0.04(0.02,0.07) | 0.08(0.05,0.13) | -1.83(-1.94,-1.73) |
| Kuwait | 0.62(0.47,0.82) | 0.06(0.04,0.07) | 4.09(1.78,6.44) |
| Kyrgyzstan | 19.96(11.85,31.83) | 0.71(0.42,1.13) | -9.16(-9.93,-8.38) |
| Lao People's Democratic Republic | 492.57(217.07,884.43) | 16.47(7.26,29.56) | -4.87(-5.05,-4.70) |
| Latvia | 0.51(0.35,0.70) | 0.13(0.09,0.18) | -9.45(-10.29,-8.61) |
| Lebanon | 1.88(1.18,2.83) | 0.11(0.07,0.17) | -4.45(-4.59,-4.31) |
| Lesotho | 37.30(21.59,62.98) | 4.45(2.57,7.51) | 0.59(0.23,0.95) |
| Liberia | 84.89(33.75,156.43) | 3.04(1.21,5.61) | -3.84(-4.22,-3.45) |
| Libya | 12.46(5.89,25.44) | 0.60(0.28,1.22) | -0.06(-0.37,0.24) |
| Lithuania | 1.03(0.72,1.39) | 0.19(0.13,0.26) | -9.43(-10.37,-8.48) |
| Luxembourg | 0.02(0.02,0.03) | 0.02(0.01,0.03) | -1.55(-2.59,-0.49) |
| Madagascar | 481.73(193.46,856.63) | 3.22(1.29,5.73) | -1.55(-1.81,-1.28) |
| Malawi | 288.75(124.02,482.37) | 2.73(1.17,4.55) | -3.46(-3.68,-3.23) |
| Malaysia | 160.18(91.48,233.34) | 1.56(0.89,2.28) | 2.36(1.13,3.61) |
| Maldives | 0.37(0.25,0.55) | 0.29(0.19,0.43) | -7.33(-7.93,-6.73) |
| Mali | 629.75(214.68,1061.52) | 4.42(1.51,7.46) | -2.04(-2.24,-1.85) |
| Malta | 0.08(0.06,0.11) | 0.10(0.07,0.13) | 0.52(-0.79,1.86) |
| Marshall Islands | 0.01(0.01,0.02) | 0.04(0.02,0.07) | -0.68(-0.80,-0.56) |
| Mauritania | 39.22(17.44,63.73) | 1.68(0.75,2.73) | -3.32(-3.67,-2.98) |
| Mauritius | 1.00(0.69,1.40) | 0.34(0.23,0.47) | 5.63(4.14,7.15) |
| Mexico | 2130.54(1684.70,2724.64) | 4.94(3.90,6.31) | 5.98(2.43,9.65) |
| Micronesia (Federated States of) | 0.02(0.01,0.03) | 0.04(0.02,0.07) | -0.96(-0.99,-0.94) |
| Monaco | 0.00(0.00,0.00) | 0.02(0.01,0.04) | -1.23(-1.49,-0.97) |
| Mongolia | 11.28(7.12,17.03) | 0.86(0.54,1.30) | -7.01(-7.56,-6.44) |
| Montenegro | 0.15(0.08,0.28) | 0.10(0.05,0.19) | -2.63(-3.13,-2.13) |
| Morocco | 127.81(48.59,280.41) | 0.99(0.38,2.17) | -2.55(-3.31,-1.78) |
| Mozambique | 877.63(320.00,1653.86) | 4.94(1.80,9.32) | -2.60(-2.86,-2.34) |
| Myanmar | 2902.48(1121.52,4720.41) | 13.98(5.40,22.73) | -4.86(-5.10,-4.61) |
| Namibia | 27.32(15.74,46.51) | 2.55(1.47,4.34) | -1.45(-1.72,-1.18) |
| Nauru | 0.00(0.00,0.00) | 0.05(0.03,0.07) | -0.64(-0.86,-0.41) |
| Nepal | 33.20(18.09,62.70) | 0.27(0.15,0.50) | -5.34(-5.50,-5.19) |
| Netherlands | 1.86(1.32,2.64) | 0.05(0.04,0.07) | 0.24(-1.05,1.54) |
| New Zealand | 0.38(0.24,0.56) | 0.03(0.02,0.04) | -0.09(-0.96,0.79) |
| Nicaragua | 12.61(5.12,23.48) | 0.49(0.20,0.90) | 0.23(-1.27,1.76) |
| Niger | 863.93(271.39,1635.30) | 5.54(1.74,10.49) | -2.89(-3.05,-2.73) |
| Nigeria | 5055.19(1943.47,7607.57) | 3.96(1.52,5.95) | -1.47(-1.80,-1.13) |
| Niue | 0.00(0.00,0.00) | 0.06(0.04,0.09) | -0.13(-0.36,0.11) |
| North Macedonia | 0.32(0.19,0.52) | 0.07(0.04,0.12) | -4.89(-5.41,-4.36) |
| Northern Mariana Islands | 0.01(0.00,0.01) | 0.04(0.02,0.07) | 0.04(-0.01,0.09) |
| Norway | 0.19(0.10,0.30) | 0.02(0.01,0.02) | -4.09(-5.19,-2.99) |
| Oman | 4.26(1.78,8.98) | 0.29(0.12,0.60) | -1.91(-2.73,-1.09) |
| Pakistan | 622.59(392.50,907.82) | 0.57(0.36,0.83) | -2.01(-2.23,-1.79) |
| Palau | 0.00(0.00,0.00) | 0.04(0.02,0.07) | -0.32(-0.37,-0.28) |
| Palestine | 8.67(5.20,13.70) | 0.36(0.22,0.57) | -1.77(-2.64,-0.89) |
| Panama | 32.13(22.63,43.68) | 2.13(1.50,2.90) | 4.87(2.27,7.53) |
| Papua New Guinea | 2.81(1.74,4.40) | 0.06(0.04,0.09) | -1.09(-1.22,-0.95) |
| Paraguay | 23.20(12.09,40.84) | 0.87(0.45,1.53) | -4.49(-4.79,-4.19) |
| Peru | 56.70(30.70,100.91) | 0.45(0.25,0.81) | -5.86(-6.80,-4.92) |
| Philippines | 6157.46(4399.98,8294.87) | 13.75(9.83,18.52) | -3.74(-3.91,-3.56) |
| Poland | 115.54(90.07,145.61) | 1.50(1.17,1.89) | 2.04(-0.44,4.59) |
| Portugal | 8.35(6.08,11.14) | 0.44(0.32,0.58) | -1.56(-3.43,0.36) |
| Puerto Rico | 8.09(5.42,11.85) | 1.24(0.83,1.81) | -2.35(-3.92,-0.75) |
| Qatar | 0.40(0.26,0.66) | 0.07(0.04,0.11) | 0.03(-1.33,1.41) |
| Republic of Korea | 5.15(3.19,8.19) | 0.06(0.04,0.10) | -3.70(-4.06,-3.34) |
| Republic of Moldova | 1.95(1.38,2.66) | 0.28(0.20,0.38) | -8.35(-9.32,-7.36) |
| Romania | 17.22(13.21,23.11) | 0.43(0.33,0.57) | -1.93(-3.12,-0.72) |
| Russian Federation | 169.89(145.55,197.29) | 0.50(0.43,0.58) | -9.76(-10.41,-9.10) |
| Rwanda | 148.45(51.14,282.28) | 2.31(0.79,4.38) | -3.69(-3.93,-3.46) |
| Saint Kitts and Nevis | 0.01(0.00,0.01) | 0.04(0.02,0.06) | 0.32(-0.11,0.75) |
| Saint Lucia | 0.04(0.03,0.07) | 0.11(0.07,0.16) | -1.32(-2.34,-0.29) |
| Saint Vincent and the Grenadines | 0.93(0.53,1.52) | 2.76(1.58,4.50) | 3.04(1.77,4.33) |
| Samoa | 0.04(0.02,0.07) | 0.04(0.02,0.07) | -0.47(-0.51,-0.43) |
| San Marino | 0.02(0.01,0.03) | 0.29(0.14,0.55) | -3.27(-3.51,-3.03) |
| Sao Tome and Principe | 1.05(0.49,2.05) | 1.03(0.48,2.01) | -5.27(-5.69,-4.86) |
| Saudi Arabia | 18.78(5.80,39.33) | 0.19(0.06,0.39) | -5.55(-5.78,-5.31) |
| Senegal | 209.11(89.53,437.14) | 2.58(1.10,5.39) | -3.38(-3.66,-3.10) |
| Serbia | 1.88(1.08,3.14) | 0.10(0.06,0.17) | -4.10(-4.68,-3.51) |
| Seychelles | 0.53(0.24,0.99) | 1.73(0.80,3.24) | -1.91(-2.36,-1.47) |
| Sierra Leone | 219.25(90.28,410.03) | 4.82(1.98,9.01) | -2.65(-2.87,-2.43) |
| Singapore | 0.53(0.33,0.86) | 0.05(0.03,0.08) | -1.10(-1.28,-0.93) |
| Slovakia | 1.59(0.94,2.86) | 0.14(0.08,0.26) | -3.80(-4.02,-3.57) |
| Slovenia | 0.34(0.25,0.48) | 0.09(0.06,0.12) | -1.75(-2.75,-0.75) |
| Solomon Islands | 0.16(0.09,0.26) | 0.05(0.03,0.08) | -1.17(-1.24,-1.10) |
| Somalia | 597.31(204.02,1215.42) | 4.67(1.60,9.51) | -1.32(-1.60,-1.04) |
| South Africa | 412.06(260.62,616.50) | 2.07(1.31,3.10) | -1.28(-1.56,-1.01) |
| South Sudan | 399.63(134.55,693.80) | 7.28(2.45,12.64) | 0.10(-0.27,0.47) |
| Spain | 7.73(5.75,10.64) | 0.09(0.07,0.12) | -0.81(-2.15,0.54) |
| Sri Lanka | 14.06(8.32,22.76) | 0.20(0.12,0.33) | -1.49(-2.21,-0.77) |
| Sudan | 432.24(215.11,828.84) | 2.02(1.01,3.88) | -1.65(-2.46,-0.83) |
| Suriname | 3.51(2.24,5.12) | 1.85(1.18,2.70) | -1.77(-2.49,-1.05) |
| Sweden | 0.66(0.37,1.07) | 0.03(0.02,0.04) | -0.05(-0.63,0.54) |
| Switzerland | 1.25(0.93,1.62) | 0.07(0.05,0.09) | -0.61(-1.79,0.58) |
| Syrian Arab Republic | 19.38(8.27,39.96) | 0.36(0.15,0.73) | -3.05(-3.41,-2.68) |
| Taiwan (Province of China) | 10.34(7.77,13.99) | 0.26(0.19,0.35) | -3.99(-4.78,-3.20) |
| Tajikistan | 78.84(35.89,144.91) | 1.77(0.81,3.25) | -4.88(-5.31,-4.45) |
| Thailand | 101.41(71.39,140.15) | 0.74(0.52,1.03) | -4.24(-4.66,-3.83) |
| Timor-Leste | 110.20(54.22,179.28) | 16.08(7.91,26.16) | -4.80(-5.18,-4.42) |
| Togo | 110.20(41.94,197.75) | 2.64(1.00,4.74) | -2.74(-2.95,-2.53) |
| Tokelau | 0.00(0.00,0.00) | 0.06(0.03,0.11) | -0.12(-0.52,0.29) |
| Tonga | 0.02(0.01,0.03) | 0.04(0.02,0.07) | -0.42(-0.51,-0.33) |
| Trinidad and Tobago | 11.37(7.33,17.10) | 3.12(2.01,4.70) | 0.70(-0.12,1.54) |
| Tunisia | 12.38(4.95,24.61) | 0.35(0.14,0.69) | -2.61(-3.04,-2.18) |
| Turkmenistan | 28.17(15.04,46.59) | 1.44(0.77,2.38) | -5.14(-5.41,-4.88) |
| Tuvalu | 0.00(0.00,0.00) | 0.04(0.02,0.07) | -1.21(-1.30,-1.12) |
| Turkey | 18.15(12.38,25.14) | 0.07(0.05,0.10) | -6.64(-6.86,-6.42) |
| Uganda | 576.93(221.81,930.89) | 2.31(0.89,3.73) | -2.24(-2.43,-2.06) |
| Ukraine | 32.63(22.03,46.15) | 0.39(0.26,0.55) | -2.00(-2.35,-1.65) |
| United Arab Emirates | 2.95(0.89,6.79) | 0.17(0.05,0.40) | -1.88(-2.62,-1.14) |
| United Kingdom | 32.61(27.40,37.23) | 0.21(0.18,0.24) | 4.10(1.33,6.94) |
| United Republic of Tanzania | 995.39(384.03,2174.92) | 3.23(1.24,7.05) | -1.13(-1.46,-0.80) |
| United States Virgin Islands | 0.02(0.01,0.03) | 0.09(0.05,0.19) | -3.89(-4.85,-2.91) |
| United States of America | 140.89(115.67,167.82) | 0.17(0.14,0.21) | 4.22(1.53,6.99) |
| Uruguay | 5.22(3.67,7.57) | 0.58(0.41,0.84) | -0.33(-1.96,1.33) |
| Uzbekistan | 329.26(196.27,517.16) | 2.61(1.56,4.10) | -3.70(-4.43,-2.96) |
| Vanuatu | 0.07(0.04,0.11) | 0.05(0.03,0.07) | -1.06(-1.20,-0.92) |
| Venezuela (Bolivarian Republic of) | 85.60(54.36,127.19) | 0.98(0.62,1.45) | 1.75(-0.06,3.60) |
| Viet Nam | 1335.36(443.72,2668.75) | 4.22(1.40,8.44) | -3.63(-3.92,-3.35) |
| Yemen | 356.47(155.01,559.25) | 2.04(0.89,3.20) | -1.98(-2.63,-1.33) |
| Zambia | 228.02(90.17,367.68) | 2.18(0.86,3.52) | -2.62(-3.03,-2.21) |
| Zimbabwe | 357.56(223.43,551.93) | 4.48(2.80,6.91) | 2.84(2.04,3.64) |

Note: EAPCs: Estimate the annual percentage change. AS-DALYs rate/100,000: age-standardized Disability-Adjusted Life Years rate (per 100,000 population).

**Table S4.** BAPC predictions of ASIR of AGN.

| ASIR | Year | low_50 | up_50 | low_60 | up_60 | low_70 | up_70 | low_80 | up_80 | low_95 | up_95 |
| --- | --- | --- | --- | --- | --- | --- | --- | --- | --- | --- | --- |
| 9.05 | 1990 | 9.04 | 9.06 | 9.03 | 9.07 | 9.03 | 9.07 | 9.03 | 9.08 | 9.01 | 9.09 |
| 8.77 | 1991 | 8.75 | 8.78 | 8.75 | 8.78 | 8.75 | 8.79 | 8.74 | 8.79 | 8.73 | 8.80 |
| 8.50 | 1992 | 8.48 | 8.51 | 8.48 | 8.51 | 8.48 | 8.52 | 8.47 | 8.52 | 8.46 | 8.53 |
| 8.25 | 1993 | 8.24 | 8.27 | 8.24 | 8.27 | 8.23 | 8.27 | 8.23 | 8.28 | 8.22 | 8.29 |
| 8.04 | 1994 | 8.03 | 8.05 | 8.03 | 8.06 | 8.02 | 8.06 | 8.02 | 8.06 | 8.00 | 8.08 |
| 7.86 | 1995 | 7.85 | 7.87 | 7.85 | 7.88 | 7.84 | 7.88 | 7.84 | 7.89 | 7.83 | 7.90 |
| 7.71 | 1996 | 7.70 | 7.72 | 7.69 | 7.73 | 7.69 | 7.73 | 7.69 | 7.73 | 7.67 | 7.75 |
| 7.58 | 1997 | 7.57 | 7.59 | 7.56 | 7.59 | 7.56 | 7.60 | 7.56 | 7.60 | 7.54 | 7.61 |
| 7.47 | 1998 | 7.46 | 7.48 | 7.46 | 7.49 | 7.45 | 7.49 | 7.45 | 7.49 | 7.44 | 7.51 |
| 7.39 | 1999 | 7.37 | 7.40 | 7.37 | 7.40 | 7.37 | 7.40 | 7.36 | 7.41 | 7.35 | 7.42 |
| 7.33 | 2000 | 7.32 | 7.34 | 7.31 | 7.34 | 7.31 | 7.35 | 7.31 | 7.35 | 7.29 | 7.36 |
| 7.31 | 2001 | 7.30 | 7.32 | 7.29 | 7.32 | 7.29 | 7.33 | 7.29 | 7.33 | 7.27 | 7.34 |
| 7.32 | 2002 | 7.31 | 7.33 | 7.31 | 7.34 | 7.31 | 7.34 | 7.30 | 7.35 | 7.29 | 7.36 |
| 7.34 | 2003 | 7.33 | 7.36 | 7.33 | 7.36 | 7.33 | 7.36 | 7.32 | 7.37 | 7.31 | 7.38 |
| 7.34 | 2004 | 7.33 | 7.35 | 7.33 | 7.36 | 7.33 | 7.36 | 7.32 | 7.37 | 7.31 | 7.38 |
| 7.30 | 2005 | 7.29 | 7.31 | 7.28 | 7.31 | 7.28 | 7.32 | 7.28 | 7.32 | 7.26 | 7.33 |
| 7.18 | 2006 | 7.17 | 7.19 | 7.16 | 7.19 | 7.16 | 7.20 | 7.16 | 7.20 | 7.15 | 7.21 |
| 7.00 | 2007 | 6.99 | 7.02 | 6.99 | 7.02 | 6.99 | 7.02 | 6.98 | 7.03 | 6.97 | 7.04 |
| 6.83 | 2008 | 6.81 | 6.84 | 6.81 | 6.84 | 6.81 | 6.84 | 6.80 | 6.85 | 6.79 | 6.86 |
| 6.68 | 2009 | 6.67 | 6.69 | 6.67 | 6.69 | 6.66 | 6.70 | 6.66 | 6.70 | 6.65 | 6.71 |
| 6.60 | 2010 | 6.59 | 6.61 | 6.59 | 6.61 | 6.58 | 6.62 | 6.58 | 6.62 | 6.57 | 6.63 |
| 6.55 | 2011 | 6.54 | 6.56 | 6.54 | 6.57 | 6.54 | 6.57 | 6.53 | 6.57 | 6.52 | 6.58 |
| 6.50 | 2012 | 6.49 | 6.51 | 6.49 | 6.52 | 6.49 | 6.52 | 6.48 | 6.52 | 6.47 | 6.54 |
| 6.46 | 2013 | 6.45 | 6.47 | 6.45 | 6.47 | 6.44 | 6.47 | 6.44 | 6.48 | 6.43 | 6.49 |
| 6.42 | 2014 | 6.41 | 6.43 | 6.41 | 6.44 | 6.41 | 6.44 | 6.40 | 6.44 | 6.39 | 6.45 |
| 6.40 | 2015 | 6.39 | 6.41 | 6.39 | 6.41 | 6.39 | 6.42 | 6.38 | 6.42 | 6.37 | 6.43 |
| 6.39 | 2016 | 6.38 | 6.40 | 6.38 | 6.41 | 6.38 | 6.41 | 6.37 | 6.41 | 6.36 | 6.43 |
| 6.39 | 2017 | 6.38 | 6.40 | 6.38 | 6.41 | 6.38 | 6.41 | 6.37 | 6.41 | 6.36 | 6.42 |
| 6.40 | 2018 | 6.39 | 6.41 | 6.39 | 6.41 | 6.38 | 6.42 | 6.38 | 6.42 | 6.37 | 6.43 |
| 6.40 | 2019 | 6.39 | 6.42 | 6.39 | 6.42 | 6.39 | 6.42 | 6.38 | 6.42 | 6.37 | 6.44 |
| 6.43 | 2020 | 6.42 | 6.44 | 6.42 | 6.44 | 6.41 | 6.45 | 6.41 | 6.45 | 6.40 | 6.46 |
| 6.41 | 2021 | 6.40 | 6.42 | 6.40 | 6.43 | 6.40 | 6.43 | 6.39 | 6.43 | 6.38 | 6.44 |
| 6.31 | 2022 | 6.20 | 6.42 | 6.17 | 6.45 | 6.13 | 6.48 | 6.09 | 6.52 | 5.98 | 6.63 |
| 6.27 | 2023 | 6.15 | 6.39 | 6.12 | 6.42 | 6.09 | 6.46 | 6.04 | 6.50 | 5.92 | 6.62 |
| 6.24 | 2024 | 6.11 | 6.37 | 6.07 | 6.40 | 6.04 | 6.44 | 5.99 | 6.49 | 5.86 | 6.62 |
| 6.20 | 2025 | 6.06 | 6.35 | 6.02 | 6.38 | 5.98 | 6.42 | 5.93 | 6.48 | 5.79 | 6.62 |
| 6.17 | 2026 | 6.01 | 6.33 | 5.97 | 6.36 | 5.92 | 6.41 | 5.87 | 6.47 | 5.71 | 6.63 |
| 6.13 | 2027 | 5.96 | 6.31 | 5.91 | 6.35 | 5.86 | 6.40 | 5.80 | 6.47 | 5.62 | 6.64 |
| 6.10 | 2028 | 5.90 | 6.29 | 5.85 | 6.34 | 5.79 | 6.40 | 5.72 | 6.47 | 5.52 | 6.67 |
| 6.06 | 2029 | 5.84 | 6.28 | 5.79 | 6.34 | 5.72 | 6.40 | 5.64 | 6.48 | 5.42 | 6.70 |
| 6.03 | 2030 | 5.78 | 6.28 | 5.72 | 6.34 | 5.64 | 6.41 | 5.55 | 6.50 | 5.30 | 6.75 |
| 5.99 | 2031 | 5.71 | 6.28 | 5.64 | 6.35 | 5.56 | 6.43 | 5.46 | 6.53 | 5.17 | 6.82 |
| 5.96 | 2032 | 5.64 | 6.28 | 5.56 | 6.36 | 5.47 | 6.45 | 5.35 | 6.57 | 5.03 | 6.89 |
| 5.93 | 2033 | 5.57 | 6.29 | 5.48 | 6.38 | 5.37 | 6.48 | 5.24 | 6.62 | 4.88 | 6.98 |
| 5.90 | 2034 | 5.49 | 6.30 | 5.39 | 6.40 | 5.27 | 6.52 | 5.12 | 6.67 | 4.71 | 7.08 |
| 5.87 | 2035 | 5.41 | 6.32 | 5.30 | 6.44 | 5.16 | 6.57 | 5.00 | 6.73 | 4.54 | 7.19 |
| 5.83 | 2036 | 5.32 | 6.34 | 5.20 | 6.47 | 5.05 | 6.62 | 4.86 | 6.80 | 4.35 | 7.32 |
| 5.80 | 2037 | 5.24 | 6.37 | 5.10 | 6.51 | 4.93 | 6.68 | 4.72 | 6.88 | 4.15 | 7.45 |
| 5.77 | 2038 | 5.14 | 6.40 | 4.99 | 6.56 | 4.81 | 6.74 | 4.58 | 6.97 | 3.95 | 7.60 |
| 5.74 | 2039 | 5.05 | 6.44 | 4.88 | 6.61 | 4.68 | 6.81 | 4.43 | 7.06 | 3.73 | 7.76 |
| 5.71 | 2040 | 4.95 | 6.47 | 4.77 | 6.66 | 4.55 | 6.88 | 4.27 | 7.16 | 3.50 | 7.92 |
| 5.68 | 2041 | 4.85 | 6.51 | 4.65 | 6.72 | 4.41 | 6.96 | 4.11 | 7.26 | 3.27 | 8.09 |
| 5.65 | 2042 | 4.75 | 6.55 | 4.53 | 6.78 | 4.27 | 7.04 | 3.94 | 7.36 | 3.04 | 8.27 |
| 5.62 | 2043 | 4.65 | 6.60 | 4.41 | 6.84 | 4.13 | 7.12 | 3.77 | 7.47 | 2.79 | 8.45 |
| 5.59 | 2044 | 4.55 | 6.64 | 4.29 | 6.90 | 3.98 | 7.20 | 3.60 | 7.59 | 2.55 | 8.64 |
| 5.56 | 2045 | 4.44 | 6.69 | 4.16 | 6.97 | 3.83 | 7.29 | 3.42 | 7.70 | 2.29 | 8.84 |
| 5.53 | 2046 | 4.33 | 6.74 | 4.03 | 7.04 | 3.69 | 7.38 | 3.25 | 7.82 | 2.04 | 9.03 |
| 5.51 | 2047 | 4.22 | 6.79 | 3.90 | 7.11 | 3.53 | 7.48 | 3.06 | 7.95 | 1.77 | 9.24 |
| 5.48 | 2048 | 4.11 | 6.84 | 3.77 | 7.18 | 3.38 | 7.57 | 2.88 | 8.07 | 1.51 | 9.44 |
| 5.45 | 2049 | 4.00 | 6.89 | 3.64 | 7.25 | 3.22 | 7.67 | 2.70 | 8.20 | 1.24 | 9.66 |
| 5.42 | 2050 | 3.89 | 6.95 | 3.51 | 7.33 | 3.07 | 7.77 | 2.51 | 8.33 | 0.97 | 9.87 |

**Table S5.** BAPC predictions of DALYs rate of AGN.

| DALYs rate | Year | low_50 | up_50 | low_60 | up_60 | low_70 | up_70 | low_80 | up_80 | low_95 | up_95 |
| --- | --- | --- | --- | --- | --- | --- | --- | --- | --- | --- | --- |
| 9.61 | 1990 | 9.60 | 9.63 | 9.60 | 9.63 | 9.59 | 9.64 | 9.59 | 9.64 | 9.57 | 9.65 |
| 9.22 | 1991 | 9.21 | 9.23 | 9.20 | 9.24 | 9.20 | 9.24 | 9.19 | 9.25 | 9.18 | 9.26 |
| 8.81 | 1992 | 8.79 | 8.82 | 8.79 | 8.82 | 8.79 | 8.83 | 8.78 | 8.83 | 8.77 | 8.84 |
| 8.28 | 1993 | 8.26 | 8.29 | 8.26 | 8.29 | 8.26 | 8.30 | 8.25 | 8.30 | 8.24 | 8.31 |
| 7.72 | 1994 | 7.70 | 7.73 | 7.70 | 7.73 | 7.70 | 7.73 | 7.69 | 7.74 | 7.68 | 7.75 |
| 7.28 | 1995 | 7.27 | 7.29 | 7.27 | 7.30 | 7.26 | 7.30 | 7.26 | 7.30 | 7.25 | 7.32 |
| 6.86 | 1996 | 6.85 | 6.87 | 6.84 | 6.87 | 6.84 | 6.88 | 6.84 | 6.88 | 6.83 | 6.89 |
| 6.50 | 1997 | 6.48 | 6.51 | 6.48 | 6.51 | 6.48 | 6.51 | 6.47 | 6.52 | 6.46 | 6.53 |
| 6.17 | 1998 | 6.16 | 6.18 | 6.15 | 6.18 | 6.15 | 6.18 | 6.15 | 6.19 | 6.14 | 6.20 |
| 5.91 | 1999 | 5.90 | 5.92 | 5.90 | 5.93 | 5.90 | 5.93 | 5.89 | 5.93 | 5.88 | 5.94 |
| 5.67 | 2000 | 5.66 | 5.68 | 5.66 | 5.68 | 5.65 | 5.69 | 5.65 | 5.69 | 5.64 | 5.70 |
| 5.43 | 2001 | 5.42 | 5.44 | 5.42 | 5.44 | 5.42 | 5.45 | 5.41 | 5.45 | 5.40 | 5.46 |
| 5.23 | 2002 | 5.22 | 5.24 | 5.22 | 5.25 | 5.22 | 5.25 | 5.22 | 5.25 | 5.21 | 5.26 |
| 5.03 | 2003 | 5.02 | 5.04 | 5.02 | 5.05 | 5.02 | 5.05 | 5.01 | 5.05 | 5.00 | 5.06 |
| 4.83 | 2004 | 4.82 | 4.84 | 4.82 | 4.84 | 4.81 | 4.84 | 4.81 | 4.85 | 4.80 | 4.86 |
| 4.67 | 2005 | 4.66 | 4.68 | 4.66 | 4.68 | 4.66 | 4.69 | 4.65 | 4.69 | 4.64 | 4.70 |
| 4.46 | 2006 | 4.45 | 4.47 | 4.45 | 4.47 | 4.45 | 4.48 | 4.44 | 4.48 | 4.43 | 4.49 |
| 4.25 | 2007 | 4.24 | 4.26 | 4.24 | 4.26 | 4.23 | 4.26 | 4.23 | 4.26 | 4.22 | 4.27 |
| 4.12 | 2008 | 4.11 | 4.12 | 4.10 | 4.13 | 4.10 | 4.13 | 4.10 | 4.13 | 4.09 | 4.14 |
| 3.97 | 2009 | 3.96 | 3.97 | 3.96 | 3.98 | 3.95 | 3.98 | 3.95 | 3.98 | 3.94 | 3.99 |
| 3.75 | 2010 | 3.75 | 3.76 | 3.74 | 3.76 | 3.74 | 3.77 | 3.74 | 3.77 | 3.73 | 3.78 |
| 3.58 | 2011 | 3.57 | 3.58 | 3.57 | 3.59 | 3.56 | 3.59 | 3.56 | 3.59 | 3.55 | 3.60 |
| 3.42 | 2012 | 3.41 | 3.42 | 3.41 | 3.43 | 3.40 | 3.43 | 3.40 | 3.43 | 3.39 | 3.44 |
| 3.28 | 2013 | 3.28 | 3.29 | 3.27 | 3.29 | 3.27 | 3.29 | 3.27 | 3.30 | 3.26 | 3.31 |
| 3.17 | 2014 | 3.16 | 3.18 | 3.16 | 3.18 | 3.16 | 3.18 | 3.15 | 3.18 | 3.15 | 3.19 |
| 3.06 | 2015 | 3.06 | 3.07 | 3.06 | 3.07 | 3.05 | 3.08 | 3.05 | 3.08 | 3.04 | 3.09 |
| 2.97 | 2016 | 2.96 | 2.98 | 2.96 | 2.98 | 2.96 | 2.98 | 2.95 | 2.98 | 2.95 | 2.99 |
| 2.86 | 2017 | 2.85 | 2.86 | 2.85 | 2.87 | 2.85 | 2.87 | 2.84 | 2.87 | 2.84 | 2.88 |
| 2.75 | 2018 | 2.74 | 2.75 | 2.74 | 2.76 | 2.74 | 2.76 | 2.73 | 2.76 | 2.73 | 2.77 |
| 2.64 | 2019 | 2.64 | 2.65 | 2.63 | 2.65 | 2.63 | 2.65 | 2.63 | 2.66 | 2.62 | 2.66 |
| 2.43 | 2020 | 2.43 | 2.44 | 2.43 | 2.44 | 2.42 | 2.44 | 2.42 | 2.45 | 2.41 | 2.45 |
| 2.30 | 2021 | 2.29 | 2.30 | 2.29 | 2.31 | 2.29 | 2.31 | 2.29 | 2.31 | 2.28 | 2.32 |
| 2.19 | 2022 | 2.15 | 2.24 | 2.13 | 2.25 | 2.12 | 2.27 | 2.10 | 2.28 | 2.06 | 2.33 |
| 2.08 | 2023 | 2.03 | 2.13 | 2.01 | 2.14 | 2.00 | 2.16 | 1.98 | 2.17 | 1.93 | 2.22 |
| 1.97 | 2024 | 1.91 | 2.02 | 1.90 | 2.03 | 1.88 | 2.05 | 1.86 | 2.07 | 1.81 | 2.13 |
| 1.86 | 2025 | 1.80 | 1.92 | 1.78 | 1.93 | 1.77 | 1.95 | 1.75 | 1.97 | 1.69 | 2.03 |
| 1.76 | 2026 | 1.69 | 1.82 | 1.68 | 1.84 | 1.66 | 1.86 | 1.63 | 1.88 | 1.57 | 1.94 |
| 1.66 | 2027 | 1.59 | 1.73 | 1.57 | 1.75 | 1.55 | 1.77 | 1.53 | 1.79 | 1.46 | 1.86 |
| 1.57 | 2028 | 1.49 | 1.64 | 1.47 | 1.66 | 1.45 | 1.68 | 1.42 | 1.71 | 1.35 | 1.79 |
| 1.48 | 2029 | 1.40 | 1.56 | 1.38 | 1.58 | 1.35 | 1.60 | 1.32 | 1.63 | 1.24 | 1.72 |
| 1.40 | 2030 | 1.31 | 1.48 | 1.29 | 1.51 | 1.26 | 1.53 | 1.23 | 1.56 | 1.14 | 1.65 |
| 1.32 | 2031 | 1.22 | 1.41 | 1.20 | 1.43 | 1.17 | 1.46 | 1.14 | 1.49 | 1.04 | 1.59 |
| 1.24 | 2032 | 1.14 | 1.34 | 1.12 | 1.37 | 1.09 | 1.39 | 1.05 | 1.43 | 0.95 | 1.53 |
| 1.17 | 2033 | 1.07 | 1.28 | 1.04 | 1.30 | 1.01 | 1.33 | 0.97 | 1.37 | 0.87 | 1.48 |
| 1.10 | 2034 | 0.99 | 1.21 | 0.97 | 1.24 | 0.93 | 1.27 | 0.89 | 1.31 | 0.78 | 1.42 |
| 1.04 | 2035 | 0.92 | 1.15 | 0.90 | 1.18 | 0.86 | 1.22 | 0.82 | 1.26 | 0.70 | 1.37 |
| 0.98 | 2036 | 0.86 | 1.10 | 0.83 | 1.13 | 0.79 | 1.16 | 0.75 | 1.21 | 0.63 | 1.33 |
| 0.92 | 2037 | 0.80 | 1.05 | 0.77 | 1.08 | 0.73 | 1.11 | 0.69 | 1.16 | 0.56 | 1.29 |
| 0.87 | 2038 | 0.74 | 1.00 | 0.71 | 1.03 | 0.67 | 1.07 | 0.62 | 1.11 | 0.49 | 1.24 |
| 0.82 | 2039 | 0.69 | 0.95 | 0.65 | 0.98 | 0.62 | 1.02 | 0.57 | 1.07 | 0.43 | 1.20 |
| 0.77 | 2040 | 0.64 | 0.91 | 0.60 | 0.94 | 0.56 | 0.98 | 0.51 | 1.03 | 0.38 | 1.17 |
| 0.73 | 2041 | 0.59 | 0.87 | 0.55 | 0.90 | 0.51 | 0.94 | 0.46 | 0.99 | 0.32 | 1.13 |
| 0.68 | 2042 | 0.54 | 0.83 | 0.51 | 0.86 | 0.47 | 0.90 | 0.42 | 0.95 | 0.27 | 1.09 |
| 0.65 | 2043 | 0.50 | 0.79 | 0.47 | 0.82 | 0.43 | 0.87 | 0.37 | 0.92 | 0.23 | 1.06 |
| 0.61 | 2044 | 0.46 | 0.75 | 0.43 | 0.79 | 0.39 | 0.83 | 0.33 | 0.88 | 0.19 | 1.03 |
| 0.57 | 2045 | 0.43 | 0.72 | 0.39 | 0.75 | 0.35 | 0.80 | 0.30 | 0.85 | 0.15 | 1.00 |
| 0.54 | 2046 | 0.39 | 0.69 | 0.36 | 0.72 | 0.31 | 0.76 | 0.26 | 0.82 | 0.11 | 0.97 |
| 0.51 | 2047 | 0.36 | 0.66 | 0.33 | 0.69 | 0.28 | 0.73 | 0.23 | 0.79 | 0.08 | 0.94 |
| 0.48 | 2048 | 0.33 | 0.63 | 0.30 | 0.66 | 0.25 | 0.70 | 0.20 | 0.76 | 0.05 | 0.91 |
| 0.45 | 2049 | 0.30 | 0.60 | 0.27 | 0.63 | 0.23 | 0.68 | 0.17 | 0.73 | 0.03 | 0.88 |
| 0.43 | 2050 | 0.28 | 0.57 | 0.24 | 0.61 | 0.20 | 0.65 | 0.15 | 0.70 | 0.00 | 0.85 |

**Table S6.** Posterior predictive checks by year for under-20 aggregated rates: observed vs model posterior predictions, 1990–2021.

| Time | val | sd | obs | low95 | up95 | cover95 | resid | zscore |
| --- | --- | --- | --- | --- | --- | --- | --- | --- |
| 1990 | 9.05124 | 0.019968 | 9.126437 | 9.012103 | 9.090378 | FALSE | 0.075197 | 3.765852 |
| 1991 | 8.766116 | 0.019569 | 8.835137 | 8.727761 | 8.804471 | FALSE | 0.069021 | 3.527089 |
| 1992 | 8.497323 | 0.019193 | 8.560628 | 8.459704 | 8.534943 | FALSE | 0.063305 | 3.29825 |
| 1993 | 8.252998 | 0.018845 | 8.311489 | 8.216062 | 8.289933 | FALSE | 0.058491 | 3.103873 |
| 1994 | 8.04083 | 0.018531 | 8.095726 | 8.00451 | 8.07715 | FALSE | 0.054896 | 2.962455 |
| 1995 | 7.862264 | 0.018251 | 7.91478 | 7.826493 | 7.898035 | FALSE | 0.052516 | 2.877485 |
| 1996 | 7.710091 | 0.017996 | 7.760828 | 7.674819 | 7.745364 | FALSE | 0.050737 | 2.81932 |
| 1997 | 7.579513 | 0.017768 | 7.628629 | 7.544688 | 7.614338 | FALSE | 0.049116 | 2.764319 |
| 1998 | 7.471353 | 0.017572 | 7.5192 | 7.436912 | 7.505793 | FALSE | 0.047848 | 2.722973 |
| 1999 | 7.38659 | 0.017412 | 7.433836 | 7.352462 | 7.420718 | FALSE | 0.047247 | 2.713422 |
| 2000 | 7.327222 | 0.017291 | 7.374651 | 7.293332 | 7.361112 | FALSE | 0.047428 | 2.742993 |
| 2001 | 7.308095 | 0.017221 | 7.356735 | 7.274342 | 7.341848 | FALSE | 0.04864 | 2.824448 |
| 2002 | 7.323286 | 0.017197 | 7.373768 | 7.289581 | 7.356992 | FALSE | 0.050482 | 2.935545 |
| 2003 | 7.343731 | 0.017187 | 7.395975 | 7.310045 | 7.377417 | FALSE | 0.052244 | 3.039775 |
| 2004 | 7.343036 | 0.017162 | 7.396367 | 7.309399 | 7.376673 | FALSE | 0.053331 | 3.107534 |
| 2005 | 7.297495 | 0.017093 | 7.350768 | 7.263993 | 7.330997 | FALSE | 0.053273 | 3.116695 |
| 2006 | 7.178311 | 0.016945 | 7.227697 | 7.145098 | 7.211523 | FALSE | 0.049386 | 2.914504 |
| 2007 | 7.004975 | 0.016738 | 7.046653 | 6.972168 | 7.037782 | FALSE | 0.041678 | 2.48999 |
| 2008 | 6.825165 | 0.016521 | 6.858327 | 6.792784 | 6.857545 | FALSE | 0.033162 | 2.007319 |
| 2009 | 6.679944 | 0.016335 | 6.706549 | 6.647927 | 6.711962 | TRUE | 0.026605 | 1.628663 |
| 2010 | 6.598879 | 0.016217 | 6.623065 | 6.567093 | 6.630664 | TRUE | 0.024186 | 1.491382 |
| 2011 | 6.553096 | 0.01613 | 6.577713 | 6.52148 | 6.584712 | TRUE | 0.024617 | 1.526101 |
| 2012 | 6.504049 | 0.016029 | 6.529285 | 6.472633 | 6.535465 | TRUE | 0.025236 | 1.574448 |
| 2013 | 6.458446 | 0.015922 | 6.484367 | 6.427238 | 6.489653 | TRUE | 0.025921 | 1.627986 |
| 2014 | 6.422378 | 0.015824 | 6.448825 | 6.391364 | 6.453392 | TRUE | 0.026447 | 1.67133 |
| 2015 | 6.401683 | 0.015742 | 6.428525 | 6.37083 | 6.432537 | TRUE | 0.026841 | 1.705122 |
| 2016 | 6.394399 | 0.015674 | 6.421553 | 6.363677 | 6.42512 | TRUE | 0.027155 | 1.732444 |
| 2017 | 6.394271 | 0.015615 | 6.421782 | 6.363665 | 6.424878 | TRUE | 0.02751 | 1.761735 |
| 2018 | 6.399199 | 0.015566 | 6.427 | 6.368689 | 6.429708 | TRUE | 0.027801 | 1.786035 |
| 2019 | 6.404577 | 0.015524 | 6.432613 | 6.37415 | 6.435005 | TRUE | 0.028036 | 1.805967 |
| 2020 | 6.429823 | 0.015519 | 6.457994 | 6.399406 | 6.46024 | TRUE | 0.028171 | 1.815252 |
| 2021 | 6.414537 | 0.015481 | 6.442443 | 6.384194 | 6.444879 | TRUE | 0.027906 | 1.802637 |

Columns: val = posterior predictive mean; sd = posterior predictive SD; obs = observed aggregated rate; low95/up95 = 95% predictive interval; cover95 = indicator that obs lies within the interval; resid = obs − val; zscore = (obs − val)/sd. Rates are per 100,000.
