# Supplementary material for: Global, regional, and national burden of acute glomerulonephritis in children and adolescents: 1990–2021 analysis and future projections
Source: Front Public Health. 2025 Dec 3;13:1677679. doi: 10.3389/fpubh.2025.1677679 (PMC12708533; doi:10.3389/fpubh.2025.1677679)
Supplement: Supplementary file 2 [file Data_Sheet_2.docx]

**

**

**Supplementary Figure S1.** Temporal trends in incidence of acute glomerulonephritis among children and adolescents in seven key countries, 1990–2021. The figure displays the age-standardized incidence rate (per 100,000 population) over time for China, Brazil, India, Laos, Timor-Leste, Mexico, and Georgia. Shaded areas represent the 95% uncertainty intervals. Data source: Global Burden of Disease Study 2021.





**Supplementary Figure S2.** Temporal trends in DALYs of acute glomerulonephritis among children and adolescents in seven key countries, 1990–2021. The figure displays the age-standardized DALYs rate (per 100,000 population) over time for China, Brazil, India, Laos, Timor-Leste, Mexico, and Georgia. Shaded areas represent the 95% uncertainty intervals. Data source: Global Burden of Disease Study 2021.

**
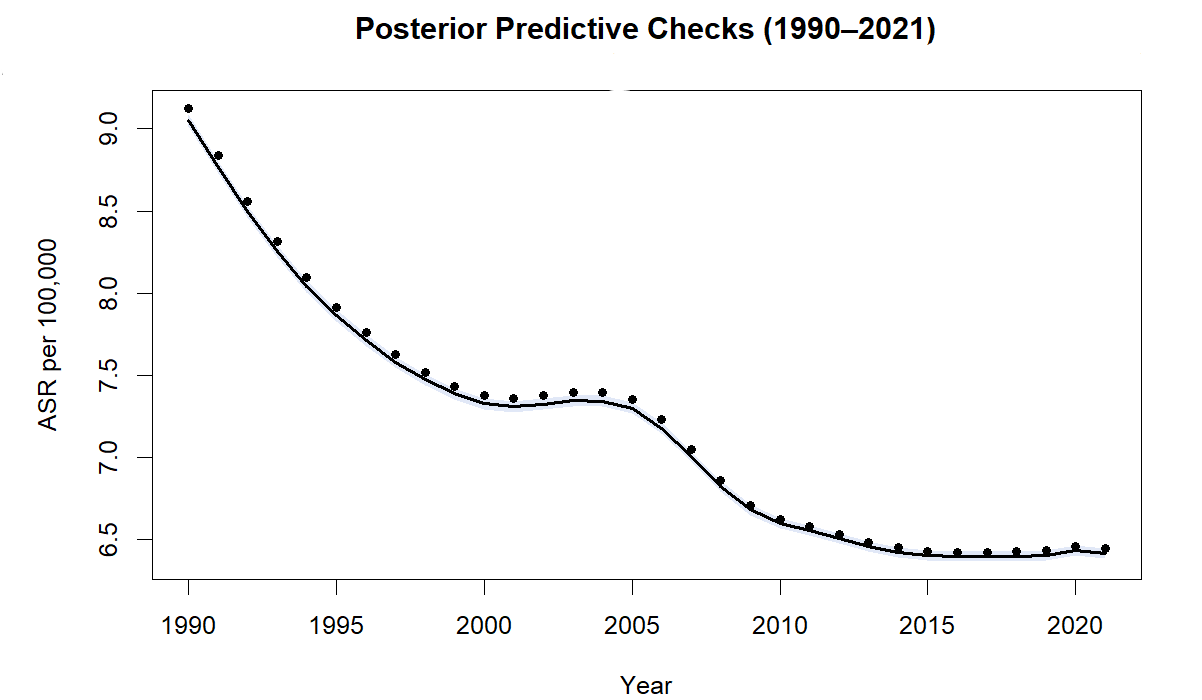
Supplementary Figure S3.** Posterior predictive check for the Bayesian Age-Period-Cohort (BAPC) model of acute glomerulonephritis in children and adolescents under 20 years, 1990-2021.
